# Supplementary material for: FeLIX is a restriction factor for mammalian retrovirus infection
Source: J Virol. 2024 Mar 5;98(4):e01771-23. doi: 10.1128/jvi.01771-23 (PMC11019853; doi:10.1128/jvi.01771-23)
Supplement: Supplemental material — Figures S1 to S15 and Tables S1 to S3. [file jvi.01771-23-s0001.pdf]

Supplementary Information

Figure legends of supplementary data

Figure S1. EnFeLV Env open reading frames (ORFs) in domestic cats. Publicly available whole-genome sequence data and genomic library screening were utilized to identify enFeLV Env ORFs in the cat genomes.

Figure S2. Recombination analysis of FeLV-B *env* genes used in this study. (A) FeLV-B and enFeLV recombinant junctions were identified using similarity plots. Plots indicate similarity between a series of highlighted sequences. Each graph is an analysis of the title sequence and the reference FeLV env sequences (color-coded). The x-axis represents the position of the env sequences, while the y-axis represents the similarity. (B) Structural representation of the various recombination structures identified using similarity plot analysis. The motifs are abbreviated as SP (signal peptide), VRA (variable region A), VRB (variable region B), PRR (proline-rich region), and C-dom. SU (surface unit) and TM (transmembrane unit). Colors indicate parts of the recombinant protein.

Figure S3. FePit1 and fePit2 expression in feline tissues and cell lines. Quantification of fePit1 and fePit2 transcripts via quantitative RT-PCR in feline tissues and cell lines. The x-axis indicates the analyzed samples, while the y-axis shows the expression level normalized to that of peptidylprolyl isomerase A (PPIA). LN, lymph node.

Figure S4. Alignment of the amino acid sequences of FeLV-A, FeLV-B strain, enFeLV, and FeLIX, Trunc-C4. The critical amino acids responsible for the receptor shift of FeLV-B, namely lysine (K) at position 66 and glutamine (Q) at position 73, are indicated by black narrow highlighting. Amino acids at positions 345 (glycine, G) and 394 (lysine, K) utilized in constructing enFeLV mutants are indicated in orange narrow, while black bold letters denote the cleavage site (**RXRR/RXKR**). Amino acid 'MGPNL' epitopes are indicated by shading blue. Conserved amino acid residues are marked by dots, gaps in the amino acid sequence by hyphens, and stop codons by asterisks. The abbreviations used are as follows: SU (surface unit), TM (transmembrane unit), VR (variable region), and PRR (proline-rich region).

Figure S5. Detection of Trunc-C4 in domestic cats and European wild cats. The viral integration of Trunc-C4 was determined via PCR in cat genomes (n = 22) and European wild cats (n = 9).

Figure S6. Inhibitory effect of the truncated Env proteins derived from enFeLV on enFeLV and FeLV-B infection. (A) Inhibition assay using FeLIX-N249 and FeLIX-D249 for the infection of Env-pseudotyped viruses, enFeLV (clone1 E345G, clone2, clone3, and AGTT), (B) FeLV-B (GA, MZ40-5B, KG20-5B, FO36-5B, and ON-T), and FeLV-B mutants (GA Q73R, FO36-5B K66D, and ON-T R73Q) in CRFK cells. (C) Dose-dependent inhibition of FeLIX for Env-pseudotyped viral infection (FeLV-B/ON-T, FeLV-B/GA, and enFeLV-AGTT) in CRFK cells. (D) Against replication-competent viruses

assessed included FeLV-B/GA, FeLV-B/ON-T, FeLV-A carrying the enFeLV-AGTT *env* gene, and FeLV-A/61E in CRFK cells. FeLIX-N249, FeLIX-D249, FeB-RBD, and the empty vector/mock were sourced from supernatants of HEK293T cells transfected with their respective expression vectors. Each supernatant was added to the culture for 2 h. Subsequently, cells were infected with the Env-pseudotyped virus. The infectious units (IU) shown on the x-axis were determined by counting the number of $\log_{10}$-galactosidase (LacZ)-positive cells per mL of virus indicated on the y-axis. Virus infection titers with standard deviations represent the means of three independent infection experiments. Comparisons were performed using Student's t-test (*$p < 0.01$).

Figure S7. Inhibitory effect of Trunc-C4 against Env-pseudotype viruses, enFeLV (clone1 E345G, clone2, clone3, and AGTT), FeLV-B (GA, MZ40-5B, KG20-5B, FO36-5B, and ON-T), and FeLV-B mutants (GA Q73R, FO36-5B K66D, and ON-T R73Q) in (A) AH927 cells and (B) CRFK cells. The infectious units (IU) were determined by counting the number of $\log_{10}$-galactosidase (LacZ)-positive cells per mL of the virus. Virus infection titers with standard deviations represented the means of three independent experiments. Mock represents the negative control. Comparisons were performed using Student's t-test (*$p < 0.01$).

Figure S8. Inhibitory effect of FeLIX on enFeLV and FeLV-B Env-pseudotype viruses in HEK293T cells as target cells. (A) Supernatants collected from HEK293T cells transfected with expression vectors encoding FeLIX-N249, FeLIX-D249, and FeB-RBD

were subjected to an inhibition assay against enFeLV (clone1 E345G, clone2, clone3, and AGTT). (B) The same supernatants were also evaluated for inhibition against FeLV-B (GA, MZ40-5B, KG20-5B, FO36-5B, and ON-T). The supernatants from HEK293T cells transfected with an empty vector/mock were utilized as a control. In each case, 250 µL of the respective supernatant was added to the cell cultures before infection with Env-pseudotyped virus. Infectious units (IU) were determined by counting the number of $\log_{10}$-galactosidase (LacZ)-positive cells per mL of virus. Virus infection titers with standard deviations represent the average of three independent experiments, with the mock serving as the negative control. Statistical comparisons were performed using Student's *t*-test (*p < 0.01).

Figure S9. Inhibitory effect of FeLIX from the supernatant of 3201 cells on FeLV-B infection with high viral titers. Inhibition assays of FeLV-B/GA in high viral titers were conducted using the culture supernatant from 3201 cells with AH927 cells as target cells. The infectious units (IU) were determined by counting the number of $\log_{10}$-galactosidase (LacZ)-positive cells per mL of the virus (x-axis). Virus infection titers with standard deviations represent the means of three independent experiments. Medium represents the negative control. Comparisons were performed using Student's *t*-test (*p < 0.01).

Figure S10. Thermal sensitivity of FeLIX. Supernatants obtained from HEK293T cells transfected with expression vectors encoding (A) FeLIX-N249 or from (B) 3201 cells were subjected to heat treatment at 56 °C for 30 min. Subsequently, 250 µL of the treated

supernatants from these cells were utilized for inhibition assays against FeLV-B infection in AH927 cells. (C) The supernatants from 3201 cells, diluted in the medium as indicated on the x-axis, were subjected to heat treatment at 56 °C for 30 min, and their effects on FeLV-T infection were assessed in AH927 cells. Infectious units (IU) were determined by quantifying the number of $\log_{10}$-galactosidase (LacZ)-positive cells per mL of virus. Viral titers are depicted as the logarithm of IU per mL with standard deviations, representing the average values derived from three independent experiments. Mock samples served as negative controls.

Figure S11. Infectivity and receptor usage of MuLV 4070A Env-pseudotyped viruses. Infectivity of MuLV 4070A Env-pseudotyped viruses in Chinese hamster ovary (CHO) cells expressing feline Pit1 (CHO-fePit1) and feline Pit2 (CHO-fePit2). Infection assay of MuLV 4070A, FeLV-B/GA, FeLV-B/ON-T, enFeLV-AGTT, and KoRV in CHO-fePit1, CHO-fePit2, and CHO-empty vector as target cells for receptor usage. The infectious units (IU) were determined by counting the number of $\log_{10}$-galactosidase (LacZ)-positive cells per mL of the virus. The viral titers are illustrated as the log number of IU per mL.

Figure S12. Inhibitory effect of Trunc-C4 on non-feline mammalian retroviruses. Inhibition assays were performed using supernatants collected from HEK293T cells transfected with Trunc-C4 expression and empty vectors against non-feline mammalian retroviruses in (A) AH927 and (B) CRFK cells. Infectious units (IU) were quantified by counting the number of $\log_{10}$-galactosidase (LacZ)-positive cells per mL of virus. The virus

infection titers, accompanied by standard deviations, represent the average values derived from three independent experiments, with mock samples used as the negative controls.

Figure S13. Alignment of the amino acid sequences of non-feline mammalian retroviruses. Conserved amino acid residues are denoted by dots, gaps in the amino acid sequence by hyphens, and stop codons by asterisks. SU, surface unit; TM, transmembrane unit; VR, variable region; PRR, proline-rich region.

Figure S14. DNA synthesis of ERV Env genes. The mammalian retrovirus Env (opt-KoRV-A2 env and opt-HPG env) in this study was synthesized by Eurofins Genomics (Tokyo, Japan).

Figure S15. Alignment of the nucleotide sequence between enFeLV-AGTT, -clone1, FeLIXs, and enFeLV-clone4. The proviral sequences with 5′ and 3′ LTRs, *gag*-*pol*, and *env* genes are shown. Dots indicate identical nucleotide sequences, and dashes indicate missing nucleotides. Nucleotide sequences encoding Env are highlighted in blue.

Table S1. Sequences of primers used in this study.

Table S2. Characteristics of enFeLV in domestic cats used in this study.

Table S3. Estimated integration time based on the substitution rate of 5′ LTR and 3′ LTR sequences.

Figure S1

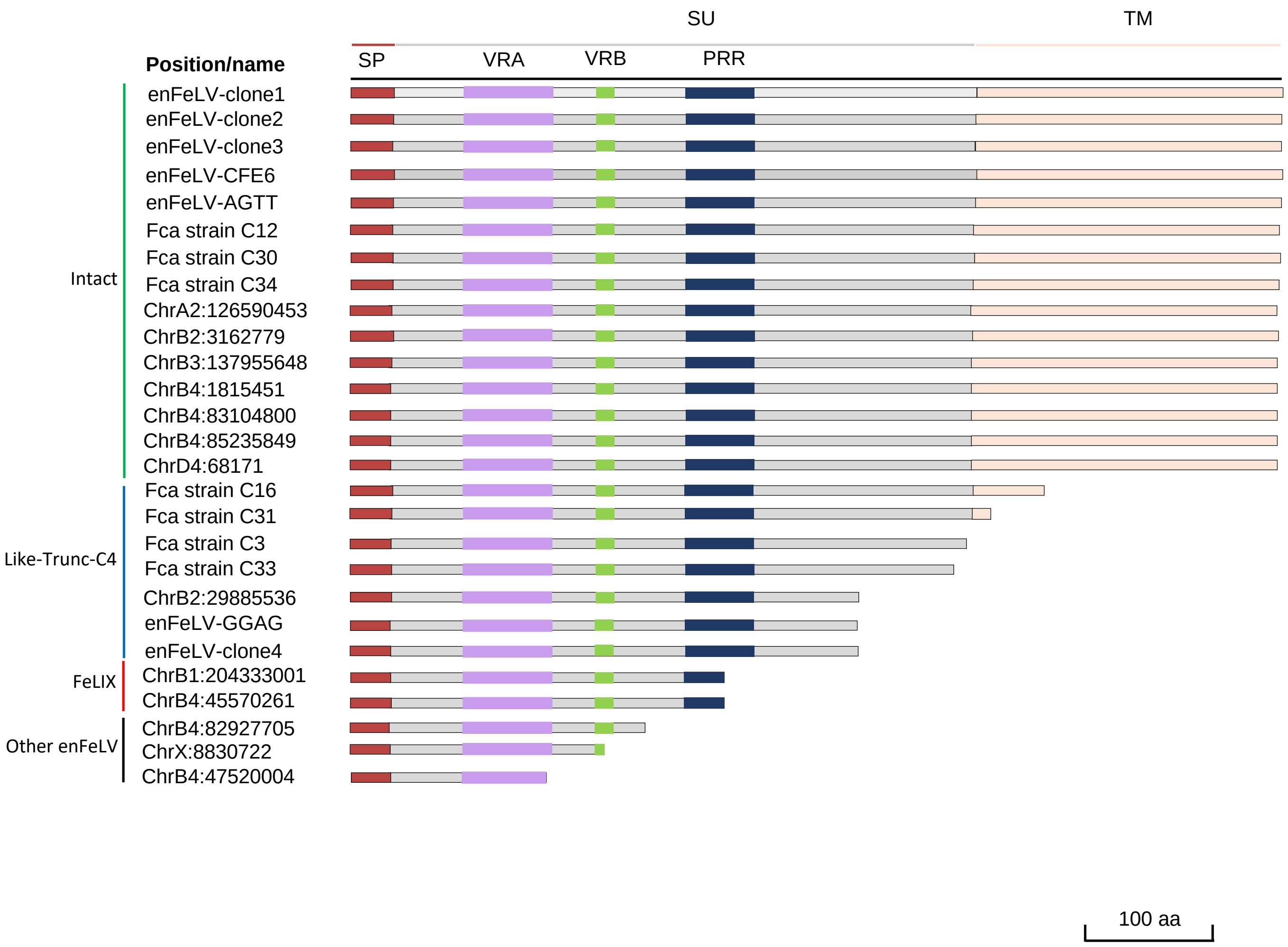

SU
TM
Position/name
SP
VRA
VRB
PRR
enFeLV-clone1
enFeLV-clone2
enFeLV-clone3
enFeLV-CFE6
enFeLV-AGTT
Fca strain C12
Fca strain C30
Fca strain C34
ChrA2:126590453
ChrB2:3162779
ChrB3:137955648
ChrB4:1815451
ChrB4:83104800
ChrB4:85235849
ChrD4:68171
Fca strain C16
Fca strain C31
Fca strain C3
Fca strain C33
ChrB2:29885536
enFeLV-GGAG
enFeLV-clone4
ChrB1:204333001
ChrB4:45570261
ChrB4:82927705
ChrX:8830722
ChrB4:47520004
Intact
Like-Trunc-C4
FeLIX
Other enFeLV
100 aa

Figure S2

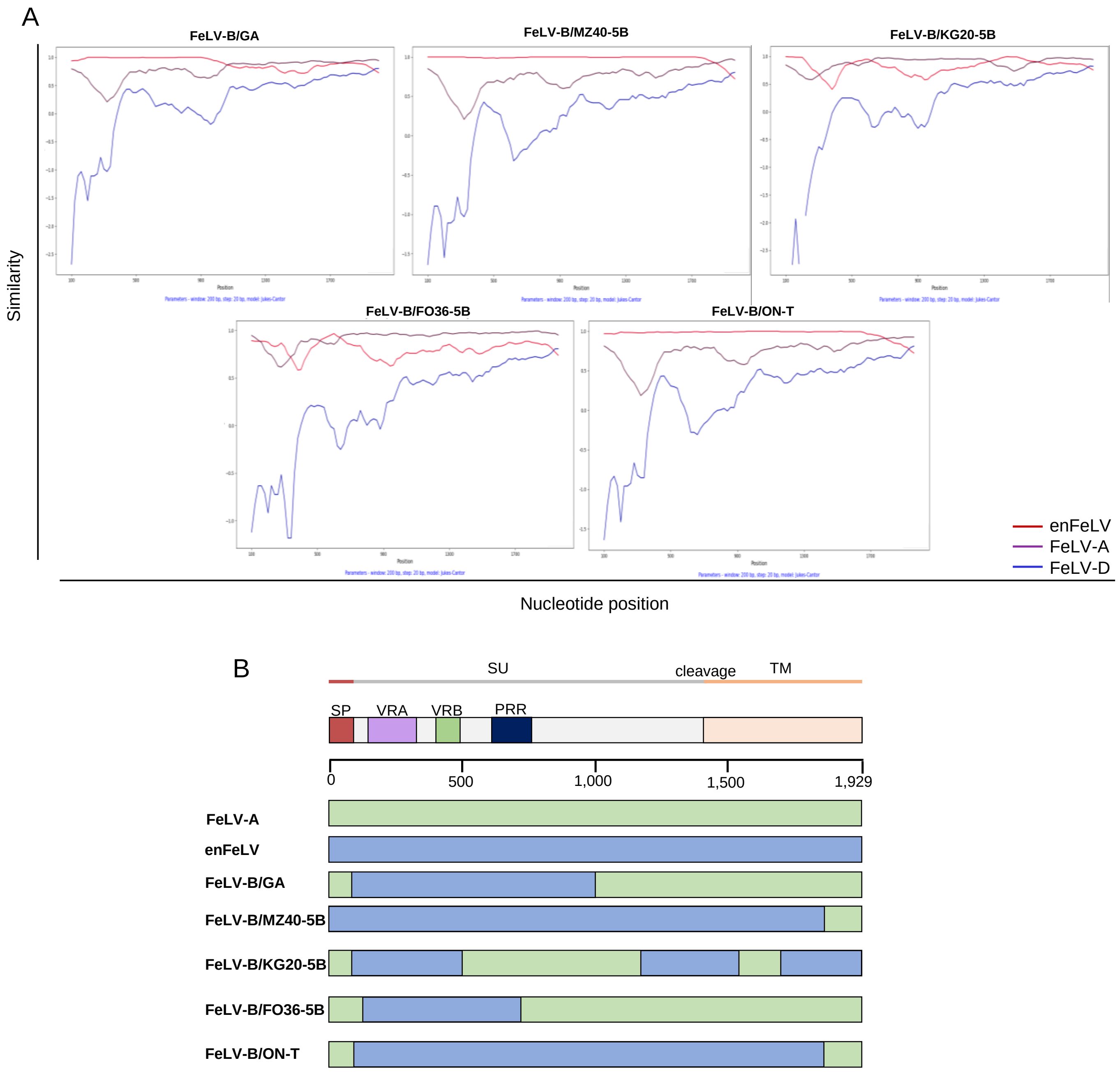

A
FeLV-B/GA
FeLV-B/MZ40-5B
FeLV-B/KG20-5B
FeLV-B/FO36-5B
FeLV-B/ON-T
Similarity
Nucleotide position
enFeLV
FeLV-A
FeLV-D
B
SU
cleavage
TM
SP
VRA
VRB
PRR
0
500
1,000
1,500
1,929
FeLV-A
enFeLV
FeLV-B/GA
FeLV-B/MZ40-5B
FeLV-B/KG20-5B
FeLV-B/FO36-5B
FeLV-B/ON-T

Figure S3

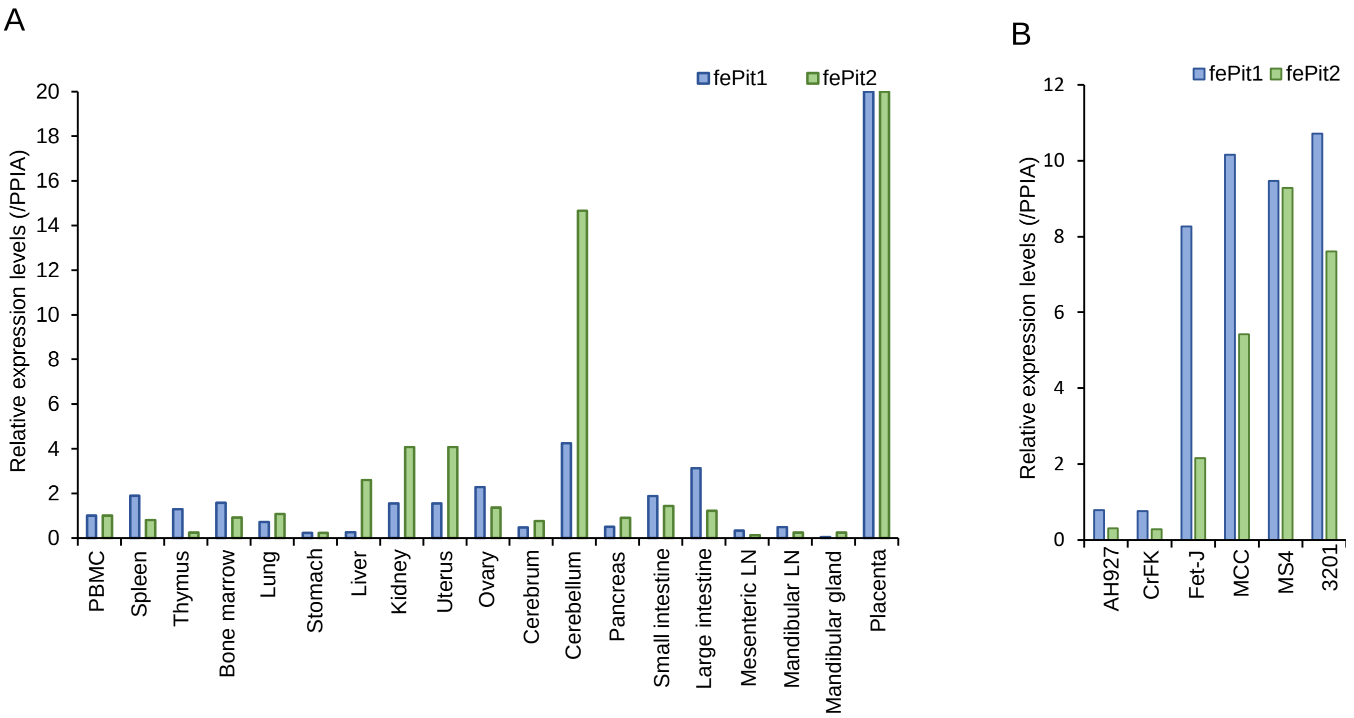

A
fePit1
fePit2
Relative expression levels (/PPIA)
20
18
16
14
12
10
8
6
4
2
0
PBMC
Spleen
Thymus
Bone marrow
Lung
Stomach
Liver
Kidney
Uterus
Ovary
Cerebrum
Cerebellum
Pancreas
Small intestine
Large intestine
Mesenteric LN
Mandibular LN
Mandibular gland
Placenta
B
fePit1
fePit2
Relative expression levels (/PPIA)
12
10
8
6
4
2
0
AH927
CrFK
Fet-J
MCC
MS4
3201

Figure S4-1

```
                        SP                  SU                                    VRA
FeLV-A/61E       1   MESPTHPKPS KDKTLSWNLV FLVGILFTID IGMANPSPHQ IYNVTWVITN VQTNTQANAT SMLGTLTDVY PTLHVDLCDL VGDTWEPIVL SPTNVKHGAR  100
FeLV-B/GA        1   .......... .......... .......... .......... V.....T... LV.G.K.... ........AF ..MYF....I I.N..N.--- -----SDQEP  92
FeLV-B/ON-T      1   ..G....... ....F..D.M ......LRV. M......... V.....T... LV.G.K.... ........AF ..MYF....I .AN..N.--- -----SDQEP  92
FeLV-B/MZ40-5B   1   ..G....... ....F..D.M I...V.LRL. V......... V.....T... LV.G.K.... ........AF ..MYF....I I.N..N.--- -----SDQEP  92
FeLV-B/KG20-5B   1   ..G....... ....F..D.M I...V.LRL. V......... V.....T... LV.G.K.... ........AF ..IYF....I I.S..N.--- -----SDQEP  92
FeLV-B/FO36-5B   1   ..G....... ....F..D.M I...V.LRL. V......... V.....T... LV.G.K.... ........AF ...YF....I I.N..N.--- -----SDQEP  92
enFeLV-AGTT      1   ..G....... ....F..D.M I...V.LRL. V......... V.....T... LV.G.K.... ........AF ..MYF....I I.N..N.--- -----SDQEP  92
enFeLV-clone1    1   ..G....... ....F..D.M I...V.LRL. V......... V.....T... LV.G.K.... ........AF ..MYF....I I.N..N.--- -----SDQEP  92
enFeLV-clone2    1   ..G....... ....F..D.M I...V.LRL. V......... V.....T... LV.G.K.... ........AF ..MYF....I I.N..N.--- -----SDQEP  92
enFeLV-clone3    1   ..G....... ....F..D.M I...V.LRL. V......... V.....T... LV.G.K.... ........AF ..MYF....I I.N..N.--- -----SDQEP  92
FeLIX-N249       1   ..G....... ....F..D.I I...V.LRL. V......... V..I..T... LV.G.K.... ........AF ...YF....I I.N..N.--- -----SGQEP  92
FeLIX-D249       1   ..G....... ....F..D.I I...V.LRL. V......... V..I..T... LV.G.K.... ........AF ...YF....I I.N..N.--- -----SGQEP  92
Trunc-C4         1   ..G....... ....F..D.M I...V.LRL. V......... V.....T... LV.G.K.... ........AF ..MYF....I I.N..N.--- -----SDQEP  92

                                                                                                   VRB
FeLV-A/61E       101 YPSSKYGCKT TDRKKQQQTY PFYVCPGHAP SLGPKGTHCG GAQDGFCAAW GCETTGEAWW KPSSSWDYIT VKRGSSQD-N NCE------- ----------  182
FeLV-B/GA        93  F.G--...DQ PM.RW..RNT ........-- ---ANRKQ.. .P......V. .......TY. R.T....... ..K.VT.GIY Q.SGGGWCGP CYDKAVHSST  185
FeLV-B/ON-T      93  F.G--...DQ PM.RWR.RNT ........-- ---ANRKQ.. .P......E. .......TY. R.T....... ....VT.GIY Q.SGGGWCGP CYDKAVHSSI  185
FeLV-B/MZ40-5B   93  F.G--...DQ PM.RW..RNT ........-- ---ANRKQ.. .P......V. .......TY. R.T....... ..K.VTKGIY Q.SGGGWCGP CYDKAVHSSI  185
FeLV-B/KG20-5B   93  F.R--...DQ PM.RW..RNT ........T. .MR....... ....Y..... ......D... ..T....... ..K.VT.GIY Q.SGGGWCGP CYDKAVHSST  190
FeLV-B/FO36-5B   93  F.G--....Q PM.RW..RNT ........N. .MR....... .......... .......... ..T....... ..K.VT.GIY Q.SGGGWCGP CYDKAVHSST  190
enFeLV-AGTT      93  F.G--...DQ PM.RW..RNT ........-- ---ANRKQ.. .P......V. .......TY. R.T....... ..K.VT.GIY Q.SGGGWCGP CYDKAVHSSK  185
enFeLV-clone1    93  F.G--...DQ PM.RW..RNT ........-- ---ANRKQ.. .P......V. .......TY. R.T....... ..K.VT.GIY Q.SGGGWCGP CYDKAVHSSI  185
enFeLV-clone2    93  F.G--...DQ PM.RW..RNT ........-- ---ANRKQ.. .P......V. .......TY. R.T....... ..K.VT.GIY Q.SGGGWCGP CYDKAVHSSI  185
enFeLV-clone3    93  F.G--...DQ PM.RW..RNT ........-- ---ANRKQ.. .P......V. .......TY. R.T....... ..K.VT.GIY Q.SGGGWCGP CYDKAVHSSI  185
FeLIX-N249       93  F.G--...DQ PM.RW..RNT A.......-- ---ANRKQ.. .P......V. .......TY. ..T....... ..K.VT.GIY Q.SGGGWCGP CYDKAVHSST  185
FeLIX-D249       93  F.G--...DQ PM.RW..RNT A.......-- ---ANRKQ.. .P......V. .......TY. ..T....... ..K.VT.GIY Q.SGGGWCGP CYDKAVHSST  185
Trunc-C4         93  F.G--...DQ PM.RW..RNT ........-- ---ANRKQ.. .P......V. .......TY. R.T....... ..K.VT.GIY Q.SGGGWCGP CYDKAVHSSI  185

                                                                                            PRR
FeLV-A/61E       182 ------GKCN PLILQFTQKG KQASWDGPKM WGLRLYRTGY DPIALFTVSR QVSTITPPQA MGPNLVLPDQ KPPSRQSQTG SKVATQRPQT N---------  267
FeLV-B/GA        186 TGASEG.R.. .......... R.T......S .......S.. ......S... ..M....... .......... ........IE .R.TPHHS.G .GGTPGITLV  285
FeLV-B/ON-T      186 TGASEG.R.. .......... R.T......S .......S.. ......S... ..M.V..... .......... .....P..IE .R.TPHHS.G .GGTPGITLV  285
FeLV-B/MZ40-5B   186 TGASEG.R.. .......... R.T......S .......S.. ......S... ..M....... .......... ........IE .R.TPHHS.G .GGTPGITLV  285
FeLV-B/KG20-5B   191 TGASEG.R.. ..V....... R......... .........R ........A. ...A...... .......... .....R..IE ......S..R H---------  281
FeLV-B/FO36-5B   191 TGASEG.R.. .......... R......... .........H .......... .......... .......... ........IE ......S..R .---------  281
enFeLV-AGTT      186 TGASEG.R.. .......... R.T......S .......S.. ......S... ..M....... .......... ........IE .R.TPHHS.G .GGTPGITLV  285
enFeLV-clone1    186 TGASEG.R.. .......... R.T......S .......S.. ......S... ..M....... .......... ........IE .R.TPHHS.G .GGTPGITLV  285
enFeLV-clone2    186 TGASEG.R.. .......... R.T......S .......S.. ......S... ..M....... .......... ........IE .R.TPHHS.G .GGTPGITLV  285
enFeLV-clone3    186 TGASEG.R.. .......... R.T......S .......S.. ......S... ..M....... .......... ........IE .R.TPHHS.G .GGTPGITLV  285
FeLIX-N249       186 TGASEG.R.. .......... R.T......S .......S.. ......S... ..M....... ....P..... ........IE .R.IPHPS- ----------  273
FeLIX-D249       186 TGASEG.R.. .......... R.T......S .......S.. ......S... ..M....... ...DP..... ........IE .R.IPHPS-- ----------  273
Trunc-C4         186 TGASEG.R.. .......... R.T......S .......S.. ......S... ..M....... .......... ........IE .R.TPHHS.G .GGTPGITLV  285

FeLV-A/61E       267 --ESAPRSVA PTTVGPKRIG TGDRLINLVQ GTYLALNATD PNKTKDCWLC LVSRPPYYEG IAILGNYSNQ TNPPPSCLSI PQHKLTISEV SGQGLCIGTV  365
FeLV-B/GA        286 NASI..L.TP V.PAS..... .......... .......... ..R....... .......... .......... .......... .......... ..........  385
FeLV-B/ON-T      286 NASI..L.TP V.PAS..... ..N....... .......V.N .......... .......... ..V....... .........D .......... ..........  385
FeLV-B/MZ40-5B   286 NASI..L.TP V.PAS..... ..N....... .......V.N .......... .......... ..V....... .........D ..Y....... ..........  385
FeLV-B/KG20-5B   282 TSSVSGPGTP -..IS...T. ......S.I. ....T....N ..I....... .......... .......... ....S....T L......... ..........  380
FeLV-B/FO36-5B   282 TSSISGPDTP -..IS...T. ......S.I. .........N .......... .......... .......... .S..S....T L......... ..........  380
enFeLV-AGTT      286 NASI..L.TP V.PAS..... ..N....... .......V.N .......... .......... ..V....... .........D .......... ..........  385
enFeLV-clone1    286 NASI..L.TP V.PAS..... ..N....... .......V.N .......... .........E ..V....... .........D .......... ..........  385
enFeLV-clone2    286 NASI..L.TP V.PAS..... ..N....... .......V.N .......... .......... ..V....... .........D .......... ..........  385
enFeLV-clone3    286 NASI..L.TP V.PAS..... ..N....... .......V.N .......... .......... ..V....... .........D .......... ..........  385
FeLIX-N249       273 ---------- ---------- ---------- ---------- ---------- ---------- ---------- ---------- ---------- ----------  273
FeLIX-D249       273 ---------- ---------- ---------- ---------- ---------- ---------- ---------- ---------- ---------- ----------  273
Trunc-C4         286 NASI..L.TP V.PAS..... ..N....... .......V.N .......... .......... ..V....... .....PI.P. RPTT------ ----------  369
```

Figure S4-2

```
                                                                                      SU         TM
FeLV-A/61E      366 PKTHQALCNK TQQGHTGAHY LAAPNGTYWA CNTGLTPCIS MAVLNWTSDF CVLIELWPRV TYHQPEYVYT HFAKAVRFRR EPISLTVALM LGGLTVGGIA 465
FeLV-B/GA       386 .........E .......... .......... .......... .......... .......... .......... .....A.... .......... .......... 485
FeLV-B/ON-T     386 ........K. ..E..K.T.. ....S..... .......... .......... .......... ...E...I.S ..ENKP..K. D......... ...I....M. 485
FeLV-B/MZ40-5B  386 ........K. ..K..K.T.. ....S..... .......... .......... .......... ...E...I.S ..ENKP..K. D......... ...I....M. 485
FeLV-B/KG20-5B  381 .......... .......... .......... .......... .......... .......... ...E...I.S ..ENKP..K. D......... ...I....M. 480
FeLV-B/FO36-5B  381 .......... .......... .......... .......... .......... .......... .......... ..D.TA.... .......... .......... 480
enFeLV-AGTT     386 ........K. ..K..K.T.. ....S..... .......... .......... .......... ...E...I.S ..ENKP..K. D......... ...I....M. 485
enFeLV-clone1   386 .......... ..K..K.T.. ....S..... .......... .......... .......... ...E...I.S ..ENKP..K. D......... ...I....M. 485
enFeLV-clone2   386 ........K. ..K..K.T.. ....S..... .......... .......... .......... ...E...I.S ..ENKP..K. D......... ...I....M. 485
enFeLV-clone3   386 ........K. ..K..K.T.. ....S..... .......... .......... .......... ...E...I.S ..ENKP..K. D......... ...I....M. 485
FeLIX-N249      273 ---------- ---------- ---------- ---------- ---------- ---------- ---------- ---------- ---------- ---------- 273
FeLIX-D249      273 ---------- ---------- ---------- ---------- ---------- ---------- ---------- ---------- ---------- ---------- 273
Trunc-C4        369 ---------- ---------- ---------- ---------- ---------- ---------- ---------- ---------- ---------- ---------- 369
                                                                                         RXRR/RXKR

FeLV-A/61E      466 AGVGTGTKAL LETAQFRQLQ MAMHTDIQAL EESISALEKS LTSLSEVVLQ NRRGLDILFL QEGGLCAALK EECCFYADHT GLVRDNMAKL RERLKQRQQL 565
FeLV-B/GA       486 .......... I......... .......... .......... .......... .......... .......... .......... .......... .......... 585
FeLV-B/ON-T     486 ..I....A.. .......... .......... ...V...... .......... .......... .......... .......... .......... .....K.... 585
FeLV-B/MZ40-5B  486 ..I....A.. .......... .......... .......... .......... .......... .......... .......... .......... .......... 585
FeLV-B/KG20-5B  481 ..I....A.. .K........ .......... V..V...R.. .......... .......... .......... .......... .......... .......... 580
FeLV-B/FO36-5B  481 .......... .......... .......... ...V...R.. .......... .......... .......... .......... ....S..... .......... 580
enFeLV-AGTT     486 ..I....A.. .......... .......... .......... .......... .......... .......... .......... .......... .......... 585
enFeLV-clone1   486 ..I....A.. .......... .......... .......... .......... .......... .......... .......... .......... .......... 585
enFeLV-clone2   486 ..I....A.. .......... .......... .......... .......... .......... .......... .......... .......... .......... 585
enFeLV-clone3   486 ..I....A.. .......... .......... .......... .......... .......... .......... .......... .......... .......... 585
FeLIX-N249      273 ---------- ---------- ---------- ---------- ---------- ---------- ---------- ---------- ---------- ---------- 273
FeLIX-D249      273 ---------- ---------- ---------- ---------- ---------- ---------- ---------- ---------- ---------- ---------- 273
Trunc-C4        369 ---------- ---------- ---------- ---------- ---------- ---------- ---------- ---------- ---------- ---------- 369

                                                                                         TM
FeLV-A/61E      566 FDSQQGWFEG WFNRSPWFTT LISSIMGPLL ILLLILLFGP CILNRLVQFV KDRISVVQAL ILTQQYQQIK QYDPDRP--- - 642
FeLV-B/GA       586 .......... ...K...... .......... .......... .......... .......... .......... .......--- - 662
FeLV-B/ON-T     586 .......... .......... .L...I...I .......... .......... .......... V......... .......--- - 662
FeLV-B/MZ40-5B  586 .......... .......... .........I .......... .......... .......... .......... ---------- - 655
FeLV-B/KG20-5B  581 .......... .......... .........I .......... .......... .......... .......... ---------- - 650
FeLV-B/FO36-5B  581 .......... .......... .........I .......... .......... .......... .......... ---------- - 650
enFeLV-AGTT     586 .......... ...K...... .........M .......... .......... ........T. V....H.RLG .C.S.Q.YHP S 666
enFeLV-clone1   586 .......... ...K...... .........M ......F... .......... ........T. V....H.RLG .C.S.Q.YHP S 666
enFeLV-clone2   586 .......... ...K...... .........M .......... .......... ........T. V...LH.RLG .C.S.Q.YHP S 666
enFeLV-clone3   586 .......... ...K...... .........M .......... .......... ........T. V....H.RLG .C.S.Q.YHP S 666
FeLIX-N249      273 ---------- ---------- ---------- ---------- ---------- ---------- ---------- ---------- - 273
FeLIX-D249      273 ---------- ---------- ---------- ---------- ---------- ---------- ---------- ---------- - 273
Trunc-C4        369 ---------- ---------- ---------- ---------- ---------- ---------- ---------- ---------- - 369
```

Figure S5

**Cat**	**Integration of Trunc-C4**	
	Positive PCR	Negative PCR
Domestic cats (n=22)	12 cases (55%)	10 cases (45%)
European wild cats (n=9)	0 cases (0%)	9 cases (100%)

Figure S6

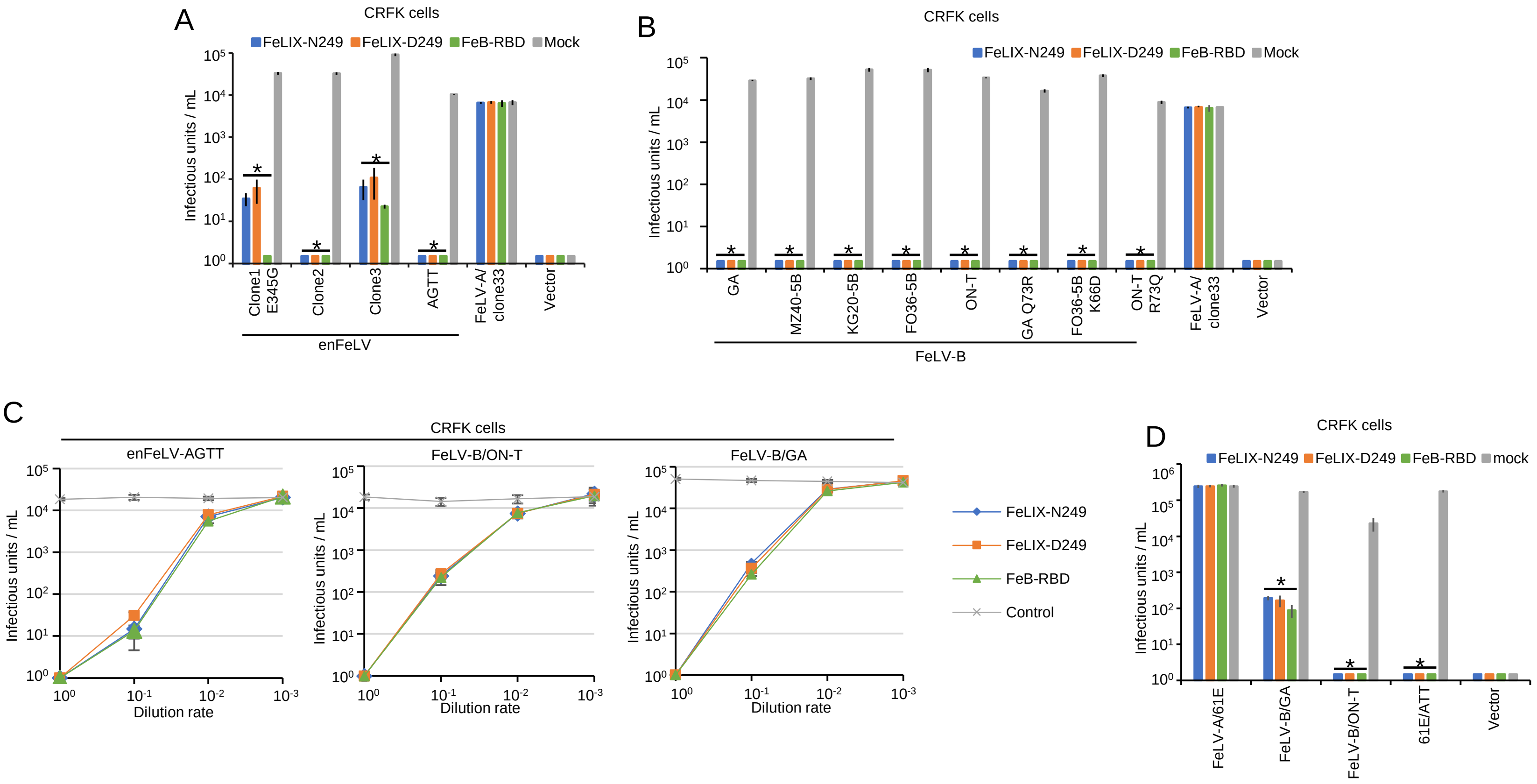
A
CRFK cells
FeLIX-N249
FeLIX-D249
FeB-RBD
Mock
Infectious units / mL
Clone1 E345G
Clone2
Clone3
AGTT
FeLV-A/ clone33
Vector
enFeLV
B
CRFK cells
GA
MZ40-5B
KG20-5B
FO36-5B
ON-T
GA Q73R
FO36-5B K66D
ON-T R73Q
FeLV-B
C
CRFK cells
enFeLV-AGTT
FeLV-B/ON-T
FeLV-B/GA
Dilution rate
Control
D
mock
FeLV-A/61E
FeLV-B/GA
FeLV-B/ON-T
61E/ATT

Figure S7

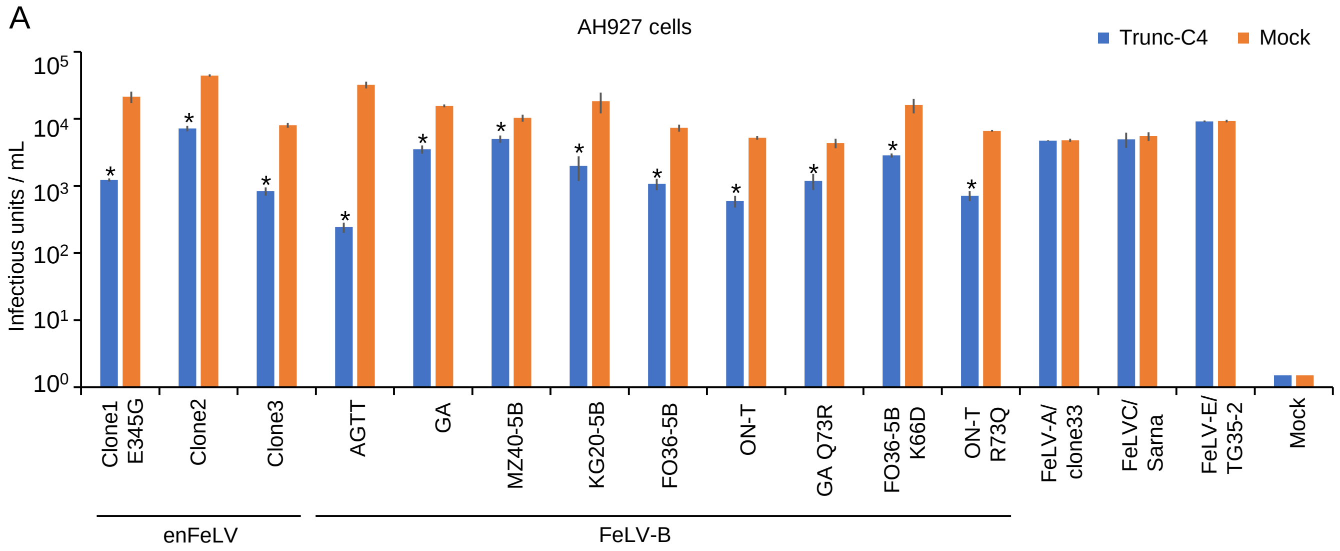
A
AH927 cells
Trunc-C4
Mock
Infectious units / mL
10^5
10^4
10^3
10^2
10^1
10^0
Clone1 E345G
Clone2
Clone3
AGTT
GA
MZ40-5B
KG20-5B
FO36-5B
ON-T
GA Q73R
FO36-5B K66D
ON-T R73Q
FeLV-A/ clone33
FeLVC/ Sarna
FeLV-E/ TG35-2
Mock
enFeLV
FeLV-B

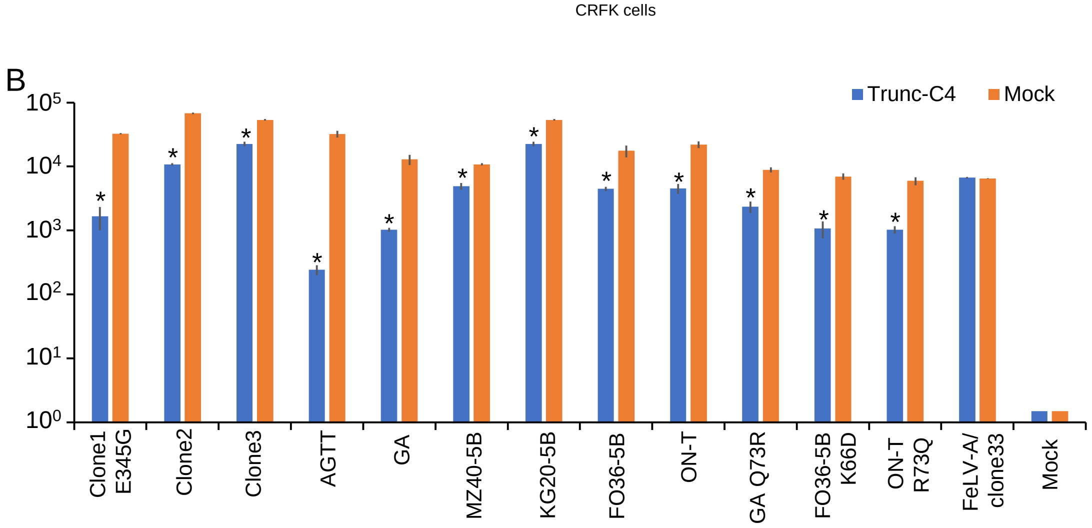
CRFK cells
B
Trunc-C4
Mock
10^5
10^4
10^3
10^2
10^1
10^0
Clone1 E345G
Clone2
Clone3
AGTT
GA
MZ40-5B
KG20-5B
FO36-5B
ON-T
GA Q73R
FO36-5B K66D
ON-T R73Q
FeLV-A/ clone33
Mock

Figure S8

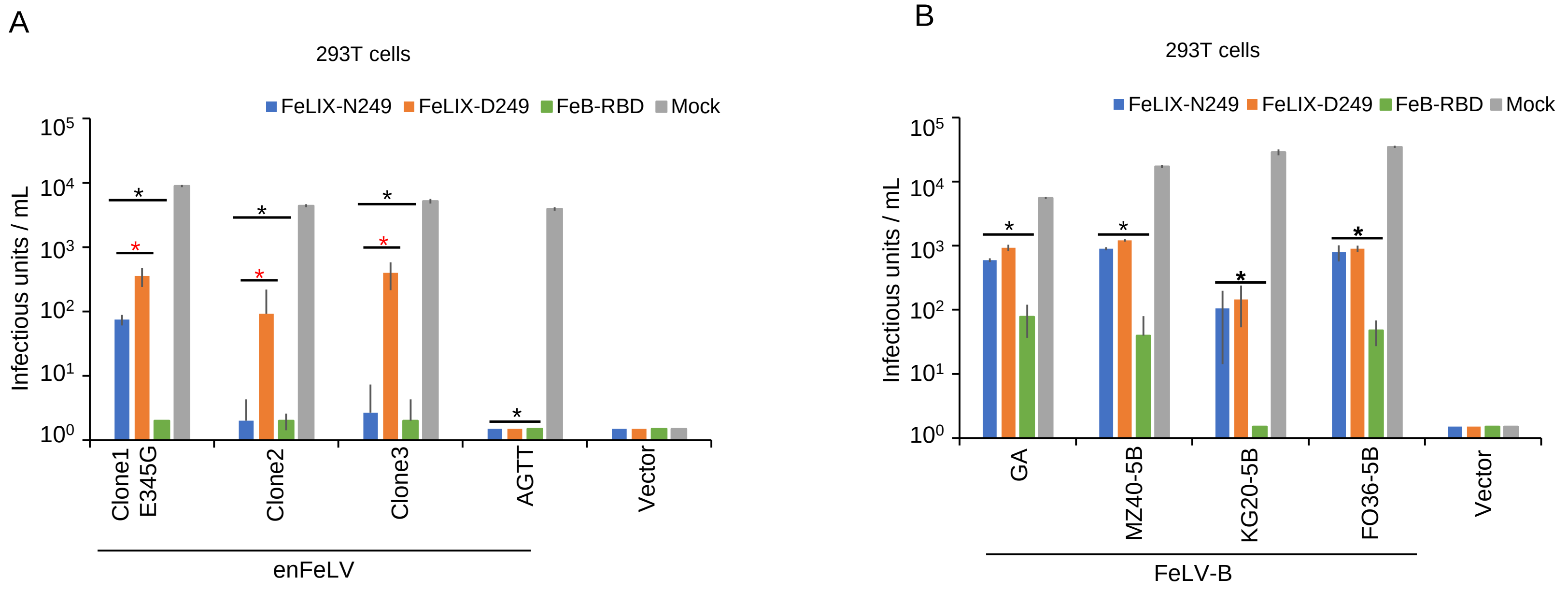
A
293T cells
FeLIX-N249
FeLIX-D249
FeB-RBD
Mock
Infectious units / mL
10^5
10^4
10^3
10^2
10^1
10^0
Clone1 E345G
Clone2
Clone3
AGTT
Vector
enFeLV
B
293T cells
FeLIX-N249
FeLIX-D249
FeB-RBD
Mock
Infectious units / mL
GA
MZ40-5B
KG20-5B
FO36-5B
Vector
FeLV-B

Figure S9

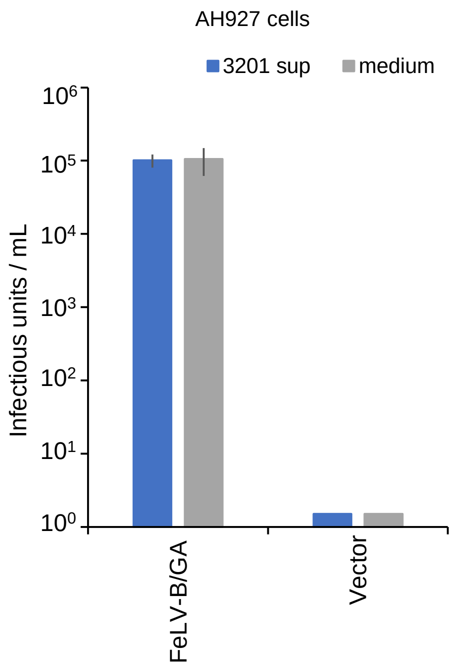

AH927 cells
3201 sup
medium
Infectious units / mL
10^6
10^5
10^4
10^3
10^2
10^1
10^0
FeLV-B/GA
Vector

Figure S10

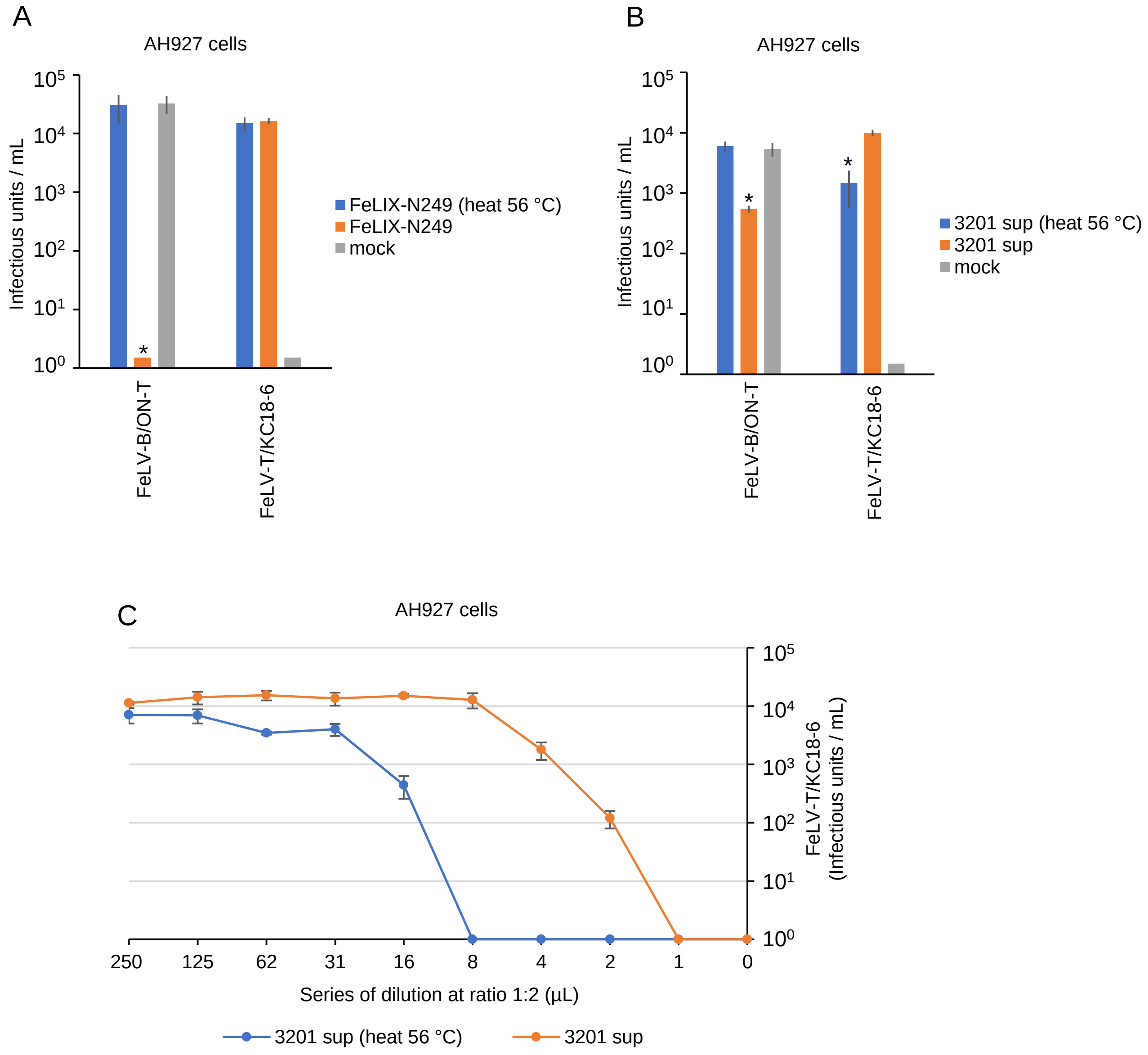
A
AH927 cells
Infectious units / mL
10^5
10^4
10^3
10^2
10^1
10^0
*
FeLV-B/ON-T
FeLV-T/KC18-6
FeLIX-N249 (heat 56 °C)
FeLIX-N249
mock
B
AH927 cells
Infectious units / mL
10^5
10^4
10^3
10^2
10^1
10^0
*
*
FeLV-B/ON-T
FeLV-T/KC18-6
3201 sup (heat 56 °C)
3201 sup
mock
C
AH927 cells
10^5
10^4
10^3
10^2
10^1
10^0
FeLV-T/KC18-6
(Infectious units / mL)
250
125
62
31
16
8
4
2
1
0
Series of dilution at ratio 1:2 (µL)
3201 sup (heat 56 °C)
3201 sup

Figure S11

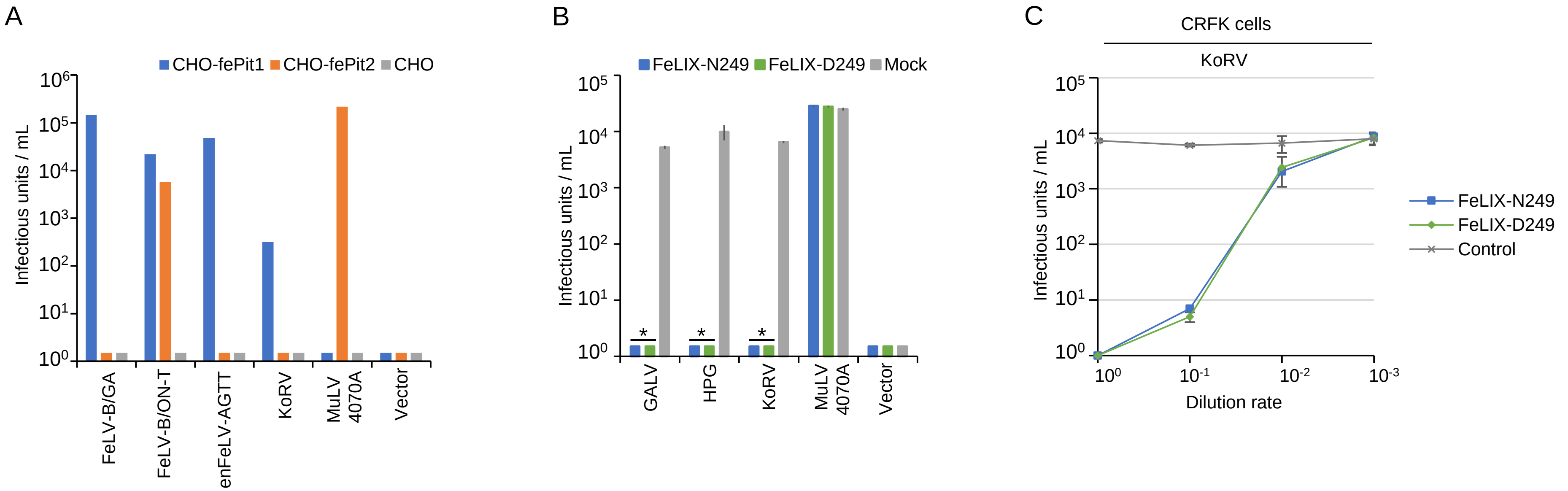
A
CHO-fePit1
CHO-fePit2
CHO
Infectious units / mL
10⁰
10¹
10²
10³
10⁴
10⁵
10⁶
FeLV-B/GA
FeLV-B/ON-T
enFeLV-AGTT
KoRV
MuLV 4070A
Vector
B
FeLIX-N249
FeLIX-D249
Mock
Infectious units / mL
*
GALV
HPG
KoRV
MuLV 4070A
Vector
C
CRFK cells
KoRV
Infectious units / mL
Dilution rate
10⁰
10⁻¹
10⁻²
10⁻³
FeLIX-N249
FeLIX-D249
Control

Figure S12

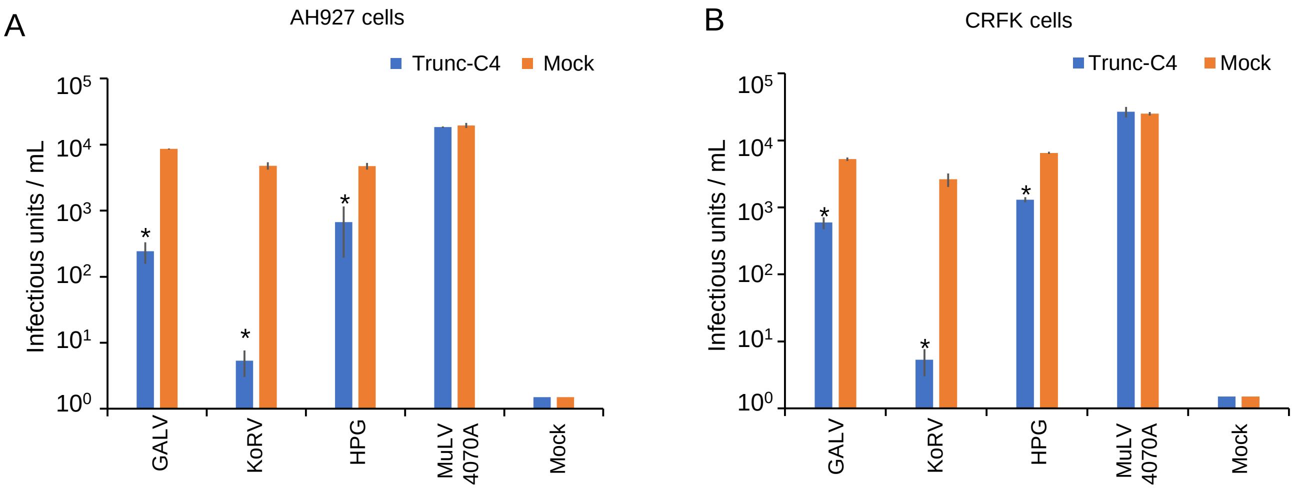
A
AH927 cells
Trunc-C4
Mock
Infectious units / mL
10^5
10^4
10^3
10^2
10^1
10^0
*
*
*
GALV
KoRV
HPG
MuLV 4070A
Mock
B
CRFK cells
Trunc-C4
Mock
Infectious units / mL
10^5
10^4
10^3
10^2
10^1
10^0
*
*
*
GALV
KoRV
HPG
MuLV 4070A
Mock

Figure S13

```
                SP                                                    SU
GALV SEATO  1   MVLLPGSMLL TSNLHHLRHQ MSPGSWKRLI ILLSCVFGGG GTSLQNKNPH QPMTLTWQVL SQTGDVVWDT KAVQPPWTWW PTLKPDVCAL AASLESWDIP  100
KoRV-A2     1   -------... I..PR..G.P ....N..... .........A EMNQ.HN... .......... ....S...EK ...E...... .S.E...... V.G.......  93
HPG         1   -------..H .LS..QP..P .N..N...V. ......L... RVKSE.N... ..V....... ....H...E. ..T..SG... .S....W... ..G..T....  93
MuLV 4070A  1   ---------- ---------- ---------- ------MARS TL.KPPQDKI N.WKPLIVMG VLL.VGMAES PHQVFNV..R V.NLMTGRTA N.TSLLGTVQ  64

                                    VRA
GALV SEATO  101 GTDVSSSKRV RPPDSDYTAA YKQITWGAIG CSYPRARTRM ASSTFYVCPR DGRTLSEARR CGGLESLYCK EWDCETTGTG YWLSKSSKDL ITVK-----W  195
KoRV-A2     94  ELTA.A.QQA .....N.EH. .N.....TL. .........I .R.Q...... ...S...... .......... ..G...A..A ..QPR..W.. ...-------  186
HPG         94  DS...A.T.. ..A....H.. T..VS...P. .......ATI .D.H...... ....P..... .......... D.G...S.SA ..HPS..W.. ...Q-----.  188
MuLV 4070A  65  DAFPKLYFDL CDLVGEEWDP SD.EPYVGY. .K..AG.Q.T RTFD.....- -.H.VKSG-- ...PGEG..G K.G.....QA ..KPT..W.. .SL.RGNTP.  160

                                 VRB                                                                    PRR
GALV SEATO  196 DQ--N----- ----SEWTQK -FQQCHQTGW CNPLKIDFTD KGKLSKDWIT GKTWGLRFYV SG-HPGVQFT I-RLKITNMP AVAVGPDLVL VEQGPPRTSL  281
KoRV-A2     186 ---------- -----GQGHP -TGT.ER... ......E..E P..RFRN.LQ .R........ T.-.....L. .-..V..SP. P.V....P.. A......K--  266
HPG         189 .N--DRRGRA SIGL.FKGHP SSNP.DNS.. .........E ...T.T...K .R.....L.R T.-.....L. .-...V.SG. TIVI...S.. A......R--  282
MuLV 4070A  161 .TGCSKVACG PCYDLSKVSN S..GATRG.R ....VLE... A..K-AN.DG P.S....L.R T.TD.ITM.S LT.QVLNVG. R.PI..NP.. PD.RL.SS--  257

GALV SEATO  282 ALPPPLPPRE APPPSLPDSN STALATSAQT PTVRKTIVTL NTPPPTTGDR LFDLVQGAFL TLNATNPGAT ESCWLCLAMG PPYYEAIASS GE-VAYSTDL  380
KoRV-A2     266 ---I.FL..- V.V.T.---- ---SPPASPI ...QASPPAP S..S...... ..G....... A......E.. ........L. .....G..TP .Q-.T.ASTD  354
HPG         282 -PS...WLYP S.S.A----- -----DNRTA L.GHT.PNP- --........ ..S....... A....D.E.. R.......L. .....G...L ..-.IPV..Q  367
MuLV 4070A  257 --.IEIV.AP Q..SP.---. TSYPPSTTS. .STSP.SPSV PQ...G.... .LA..K..YQ A..L...DK. QE.....VS. .....GV.VV .TYTNH..AP  352

GALV SEATO  381 DRCRWG-TQG KLTLTEVSGH GLCIGKVPFT HQHLCNQTLS INSSGDHQYL LPSNHSWWAC STGLTPCLST SVFNQTRDFC IQVQLIPRIY YYPEEVLLQA  479
KoRV-A2     355 SQ....-GK. .........L ........P. ......L.IP L.A.HT.K.. .......... NS........ .....SN... ..I..V.... .H.DGT....  453
HPG         368 AQ....AAR. .........R ........S. .........A L.F.QS.... R...Y...S. N......I.. .I...SK... V.......V. .HSD.A.M..  467
MuLV 4070A  353 AN.T-ATS.H ....S..T.Q ...M.A..K. ..A...T.-- QSAGSGSY.. AAPAGTM... .......... T.L.L.T.Y. VL.E.W..VI .HSPDYMYGQ  449

                SU        TM
GALV SEATO  480 YDNSHPRTKR EAVSLTLAVL LGLGITAGIG TGSTALIKGP IDLQQGLTSL QIAIDADLRA LQDSVSKLED SLTSLSEVVL QNRRGLDLLF LKEGGLCAAL  579
KoRV-A2     454 .ESP...N.. .P........ ....VA.... .......... .......... ...M.T.... ....I..... .......... .......... ..........  553
HPG         468 F-TP...L.. .P......A. .........S ...A...... ....S..AD. .A...T..Q. ....I....S .......... .......... ..........  566
MuLV 4070A  450 L-EQRTKY.. .P......L. ..GLTMG..A A.IGTGTTAL .KT..-FEQ. HA..QT..NE VEK.ITN..K .......... .......... ..........  547

GALV SEATO  580 KEECCFYIDH SGAVRDSMKK LKEKLDKRQL ERQKSQNWYE GWFNNSPWFT TLLSTIAGPL LLLLLLLILG PCIINKLVQF INDRISAVKI LVLRQKYQAL  679
KoRV-A2     554 .......V.. ........RR ...R...... .H..NLS... ....R...L. ....AL.... .......T.. ..V....... ....V...R. ....H...T.  653
HPG         567 .......V.. .....E..N. ...R.NA... ....T.S... ....S...Y. ....A..... .......TF. .....R.... ....VG.... ........V.  666
MuLV 4070A  548 .......A.. T.L.....A. .R.R.NQ..K LFETG.G.F. .L..R..... ..I...M... IV...I.LF. ...L.R.... VK....V.QA ...T.Q.HQ.  647

                TM
GALV SEATO  680 ENEGNL- 685
KoRV-A2     654 D..D..- 659
HPG         667 D..D..- 672
MuLV 4070A  648 KPIEYEP 654
```

Figure S14

KoRV-A

```
  1 MLLISNPRHL GHPMSPGNWK RLIIILLSCVF GGAEMNQQHN NPHQPMTLTW QVLSQTGSVV WEKKAVEPPW TWWPSLEPDV 80
 81 CALVAGLESW DIPELTASAS QQARPPDSNY EHAYNQITWG TLGCSYPRAR TRIARSQFYV CPRDGRSLSE ARRCGGLESL 160
161 YCKEWGCETA GTAYWQPRSS WDLITVGQGH PTGTCERTGW CNPLKIEFTE PGKRFRNWLQ GRTWGLRFYV TGHPGVQLTI 240
241 RLVITSPPPV VVGPDPVLAE QGPPRKIPFL PRVPVPTLSP PASPIPTVQA SPPAPSTPSP TTGDRLFGLV QGAFLALNAT 320
321 NPEATESCWL CLALGPPYYE GIATPGQVTY ASTDSQCRWG GKGKLTLTEV SGLGLCIGKV PPTHQHLCNL TIPLNASHTH 400
401 KYLLPSNHSW WACNSGLTPC LSTSVFNQSN DFCIQIQLVP RIYYHPDGTL LQAYESPHPR NKREPVSLTL AVLLGLGVAA 480
481 GIGTGSTALI KGPIDLQQGL TSLQIAMDTD LRALQDSISK LEDSLTSLSE VVLQNRRGLD LLFLKEGGLC AALKEECCFY 560
561 VDHSGAVRDS MRRLKERLDK RQLEHQKNLS WYEGWFNRSP WLTTLLSALA GPLLLLLLLL TLGPCVINKL VQFINDRVSA 640
641 VRILVLRHKY QTLDNEDNL 659
```

HPG

```
  1 MLHTLSLHQP RHPMNPGNWK RVIILLSCVL GGGRVKSENN NPHQPVTLTW QVLSQTGHVV WETKATQPSG TWWPSLKPDW 80
 81 CALAAGLETW DIPDSDVSAS TRVRPADSDY HAATKQVSWG APGCSYPRAR ATIADSHFYV CPRDGRTPSE ARRCGGLESL 160
161 YCKDWGCETS GSAYWHPSSS WDLITVQWDN DRRGRASIGL SFKGHPSSNP CDNSGWCNPL KIDFTEKGKT STDWIKGRTW 240
241 GLRLYRTGHP GVQLTIRLKV TSGPTIVIGP DSVLAEQGPP RRPSPPLWLY PSPSPADNRT ALTGHTTPNP PPPTTGDRLF 320
321 SLVQGAFLAL NATDPEATRS CWLCLALGPP YYEGIASLGE VIPVTDQAQC RWGAARGKLT LTEVSGRGLC IGKVPSTHQH 400
401 LCNQTLALNF SQSHQYLRPS NYSWWSCNTG LTPCISTSIF NQSKDFCVQV QLIPRVYYHS DEALMQAFTP HPRLKREPVS 480
481 LTLAALLGLG ITAGISTGSA ALIKGPIDLQ SGLADLQAAI DTDLQALQDS ISKLESSLTS LSEVVLQNRR GLDLLFLKEG 560
561 GLCAALKEEC CFYVDHSGAV RESMNKLKER LNARQLERQK TQSWYEGWFN SSPWYTTLLS AIAGPLLLLL LLLTFGPCII 640
641 NRLVQFINDR VGAVKILVLR QKYQVLDNED NL 672
```

Figure S15-1

```
enFeLV-AGTT       1    TGAAAGACCC CTTCCCCTTG TTTTGACCCC CTGTCATAAT ATGCTTAGCA ATAGTAACGC CATTTGCAAG ACAGCACCAA GAAGTTCAGG  90
enFeLV-clone1     1    .......... .......... .......... .......... .......... .......... .......... .......... ..........  90
ChrB1:204342744   1    ......T... .......... C......... .......... .......... .......... .......... G........G ..........  90
ChrB4:45570261    1    ......T... .......... C......... .......... .......... .......... .......... G........G ..........  90
enFeLV-clone4     1    .......... .......... .......... .......... .......... .......... .......... .......... ..........  90

enFeLV-AGTT       91   GGTCTTATCC TAAGTCCACC GTTTAGCTGC CAAACAGGAT ATCTGTGGTC AGCCACCCGG CCCTAAGATA GCCACCTGGC CCTAAGATGG  180
enFeLV-clone1     91   .......... .......... .......... .......... .......... .......... .......... .......... ..........  180
ChrB1:204342744   91   .........T ......A... .......... .......... .......... .......T.. .......... .GA.-----G GGA...TACT  175
ChrB4:45570261    91   .........T .......... .......... .......... .......... .......T.. .......... .GA.-----. GGA...TACT  175
enFeLV-clone4     91   .......... .......... .......... .......... .......... .......... .......... .......... ..........  180

enFeLV-AGTT       181  GAATGGAAAG TACTGACTCC ACCCGATAGA CCCTAGAGA- TGAGCCTAGT CAG--CCACC CATGTTTTTC CCCCTCATTC TGGGAAATCG  267
enFeLV-clone1     181  .......... .......... .......... .........- .......... ...--..... .......... .......... ..........  267
ChrB1:204342744   176  ..C.CC.CCC G.TAT..-.. TAGA...-.. G..AT.TC.G CC.T..AT.. TT.TT..C.T ...TC.GGAA AAT.A.CC.. A..-------  256
ChrB4:45570261    176  ..C.CC.CCC G.TAT..-.. TAGA...-.. G...G.TC.G CC.C..AT.. TT.TT..C.T ...TC.GGAA AAT.A.CC.. A..-------  256
enFeLV-clone4     181  .......... .......... .......... .........- .......... ...--..... .......... .......... ..........  267

enFeLV-AGTT       268  CCCTCAGAAA AGAAAAGAAA AAGAAAAAAA AAAAAAAAAA AAAAAAAACC AGCCTCATTT AACTGGACCA ATAAGACCCC GTAACTATGC  357
enFeLV-clone1     268  .......... .......--- ---------G .......... .......... .......... .......... .......... ..........  345
ChrB1:204342744   256  ---------- ---------- ---------- ---------. .......C.A GC.TCAT..A ..TG.AC.A. TA.GA..... .....C....  307
ChrB4:45570261    256  ---------- ---------- ---------- ---------. .......C.A GC.TCAT..A ..TG.AC.A. TA.GA..... .....C....  307
enFeLV-clone4     268  .......... .......--- ---------- -.G....... .......... .......... .......... .......... ..........  343

enFeLV-AGTT       358  TTCTCGCTTC TGTAACCGCG CTTCTGCCAC TCCAACCCTA TAAAAAGTCT CCCCAGCCCA -ACAAGAGGC GCGCAAGTCT TTGCTGAGAC  446
enFeLV-clone1     346  .......... .......... .......... .......... .......... .......... -......... ..A....... ..........  434
ChrB1:204342744   308  .......... .......... .......... .......... .....GTCTC ...AGC..A. C.GG..GC.. ..AAGTC.T. GCTGA..CTT  397
ChrB4:45570261    308  .......... .......... .......... .......... .....GTCTC ...AGC..A. C.GG..GC.. ..AAGTC.T. GCTGA..CTT  397
enFeLV-clone4     344  .......... .......... .......... .......... .......... .......... -......... .......... ..........  432

enFeLV-AGTT       447  TTGACCGCCC CGGGTACCCG TGTA-CGAAT AAACCTCTTG CTGTTTGCAT CTGACTCGTG GTCTCGGTGT TCCGTGGGCA CGGGGTCTCA  535
enFeLV-clone1     435  .......... .......... ....-..... .......... .......... .......... .......... .......... ..........  523
ChrB1:204342744   398  GACCA.A... .......... ....C..... .......... .......... .......... .......... .......... ..........  487
ChrB4:45570261    398  GAC--..... .......... ....C..... .......... .......... .......... .......... .......... ..........  485
enFeLV-clone4     433  .......... T........A ....-..... .......... .......... .......... .......... .......... ..........  521

                                               5'LTR
enFeLV-AGTT       536  TCGCCGAGGA AGACCTAGTT AGGGGGTCTT TCATTTGGGG GCTCGTCCGG GATAGAGACC CCCAACCCCC GGGACCACCG ACCCACCATC  625
enFeLV-clone1     524  .......... .......... C......... .......... .......... .......... .......... .......... ..........  613
ChrB1:204342744   488  .......... .......... CT........ .......... .......... .......... .......... .......... ..........  577
ChrB4:45570261    486  .......... .......... CT........ .......... .......... .......... .......... .......... ..........  575
enFeLV-clone4     522  .......... .......... C......... .......... .......... .......... .......... .......... ..........  611

enFeLV-AGTT       626  AGGAGGTAAG CTGGCCGGCG ACCATATCTG TTGTCCTTGT ATGAGTGTCT CTGTCATTTG ATCTGATTTT GGCGGTGGAG CCGAAGGAGC  715
enFeLV-clone1     614  G......... .......... .......... .......... .......... .......... .......... .......... ..........  703
ChrB1:204342744   578  .......... .......... ....G..... .......... .......... .......... .......C.. .......... T.........  667
ChrB4:45570261    576  .......... .......... ....G..... .......... .......... .......... .......C.. .......... T.........  665
enFeLV-clone4     612  .......... .......... .......... .......... .......... .......... .......... .......... ..........  701
```

Figure S15-2

```
enFeLV-AGTT        716  TGACGAGCTC GAACTTCGCC CCCGCAACCC TGGAAGACGT TCCACGGGTG TCTGATGTCT GGAGCCTCTA GTGGGACAGC CATTGGGGCT  805
enFeLV-clone1      704  .......... .......... .......... .......... .......... .......... .......... .......... ..........  793
ChrB1:204342744    668  .......... A......... .......... .......... .......... .......... .......... .......... ..........  757
ChrB4:45570261     666  .......... A......... .......... .......... .......... .......... .......... .......... ..........  755
enFeLV-clone4      702  .......... .......... .......... .......... .......... .......... .......... .......... ..........  791

enFeLV-AGTT        806  AGATTGTTTG GGATCTCATC CGTATTAGGT GAATACAGGG TGTTGATCGG AGACGAGGGA GCCGGACCCT CAAGGTCTCC TTCTGAGGTT  895
enFeLV-clone1      794  .......... .......... .......... .......... .......... .......... .......... .......... ..........  883
ChrB1:204342744    758  .A...T.... .......... .....C.... .......... .......... .......... .......... ...A...... ..........  847
ChrB4:45570261     756  .A...T.... .......... .....C.... .......... .......... ....A..... .......... ...A...... ..........  845
enFeLV-clone4      792  .......... .......... .......... .......... .......... .......... .......... .......... ..........  881

enFeLV-AGTT        896  TCATTTTCGG TTTGGTATCG AAGCCGCGCG GCACGTCTTG TCATTCTTTG TCTTGTTGCG TCTTTCCTTG TCCCCTGTCT AACCTTTTTA  985
enFeLV-clone1      884  .......... .......... .......... .......... .......... .......... .......... .......... ..........  973
ChrB1:204342744    848  .......... .........A .......... .......... .......... ......CA.. .......... .......... ..........  937
ChrB4:45570261     846  .......... .........A .......... .......... .......... ......CA.. .......... .......... ..........  935
enFeLV-clone4      882  .......... .......... .......... .......... .......... .......... .......... .......... ..........  971

                                       gag-pol
enFeLV-AGTT        986  ATTGCAGAAG CCGTCATGGG CCAAACTGTA ACTACCCCCT TGAGCCTCAC CCTCAACCAC TGGTCTGAGG TCCAGGCACG GGCCCGTAAT  1075
enFeLV-clone1      974  .......... .......... .......... .......... .......... .......... .......... .......... ..........  1063
ChrB1:204342744    938  .......... .......... .......... .......... .......... ....G..... .....C.... .T.G...... A....A....  1027
ChrB4:45570261     936  .......... .......... .......... .......... .......... ...TG..... .....C.... .T.G...... A....A....  1025
enFeLV-clone4      972  .......... .......... .......... .......... .......... .......... .......... .T........ ..........  1061

enFeLV-AGTT        1076 CAGGGTGTCG AAGTCCGGAA AAAGAAATGG ATTACACTGT GTGAAGCCGA ATGGGTAATG ATGAATGTAG GTTGGCCCCG AGAAGGAACT  1165
enFeLV-clone1      1064 .......... .......... .......... .......... .......... .......... .......... .......... ..........  1153
ChrB1:204342744    1028 .......... .......... .......... .......... ....G..... .......... .......... .......... ..........  1117
ChrB4:45570261     1026 .......... .......... .......... .......... ....G..... .......... .......... .......... ..........  1115
enFeLV-clone4      1062 .......... .......... .......... .......... .......... .......... .......... .......... ..........  1151

enFeLV-AGTT        1166 TTCACCATTG ACAATATTTC ACAGGTCGAG GAGAGAATCT TCG-CCCCGG GGCCATATGG ACACCCAGAT CAAATCCCTT ATATTACCAC  1254
enFeLV-clone1      1154 .......... .......... .......... .......... ...-...... .......... .......... .......... ..........  1242
ChrB1:204342744    1118 .......... .......... .......... ......G... ...-...... .......... .......... ...G...... ..........  1206
ChrB4:45570261     1116 .......... .......... .......... ......G... ...C...... .......... .......... ...G...... ..........  1205
enFeLV-clone4      1152 .......... .......... .......... .......... ...-...... .......... .......... .......... ..........  1240

enFeLV-AGTT        1255 GTGGAGATCC CTAGCCACAG ACCCCCCTCC ATGGGTTCGC CCATTCCTGC CCCCTCCTAA GCATCCCAGG ACAGATCCTC CCG-------  1337
enFeLV-clone1      1243 .......... .....T.... .......... .......... .......... .......... .......... .......... ...-------  1325
ChrB1:204342744    1207 .......... T.G.------ ----...... .......... ..G....... .......... .......... ..G....... ...CGCCACT  1286
ChrB4:45570261     1206 .......... T.G....... .......... .......... ..G....... .......... .......... .TG....... ...CGCCACT  1295
enFeLV-clone4      1241 .......... .......... .......... .......... .......--- ---------- ---------- ---------- ----------  1287

enFeLV-AGTT        1337 -----AGCCT CTTTCGCCGC AACCCTCAGC CCCCCCCATC TCCTCCCTCT ACCCCGTTCT CCCCAAACCA GACCCCCCCA AGGCGCCTGT  1422
enFeLV-clone1      1325 -----..... .......... .......... .......... .......... .......... .......... .......... ..........  1410
ChrB1:204342744    1287 TCCCC..... .......... .....C.... .......... .......... .......... .......... .......... ..........  1376
ChrB4:45570261     1296 TCCCC..... .......... .......... .......... .......... .......... .......... .......... ..........  1385
enFeLV-clone4      1287 ---------- ---------- ---------- ---------- ---------- ---------- ---------- ---------- ----------  1287
```

Figure S15-3

```
enFeLV-AGTT          1423 ATTACCACCC AATCCTTCTT CCCCTTTAAT TGATCTCTTA ACAGAAGAGC CACCTCCCTA TCCTGGGGGT CACGGGCCAA CACCGCCGTC  1512
enFeLV-clone1        1411 .......... .......... .......... .......... .......... .......... .......... .......... ..........  1500
ChrB1:204342744      1377 .......... G.....C... .......... .......... .......... .......... .......... .......... ..........  1466
ChrB4:45570261       1386 .......... G.....C... .......... .......... .......... .......... .......... .......... ..........  1475
enFeLV-clone4        1287 ---------- ---------- ---------- ---------- ---------- ---------- ---------- ---------- ----------  1287

enFeLV-AGTT          1513 AGGCCCTAGA ACCCCAACTG CCTCCCCGAT TGCCAGCCGG CTGCGAGAAC GACGAGAAAA TCCAGCTGAG AAATCTCAAG CCCTCCCCTT  1602
enFeLV-clone1        1501 .......... .......... .......... .......... .......... .......... .......... .......... ..........  1590
ChrB1:204342744      1467 ......C... .......... .......A.. .......... .......... .......... .......... G......... ..........  1556
ChrB4:45570261       1476 ......C... .......... .......A.. .......... .........T .......... .......... G......... ..........  1565
enFeLV-clone4        1287 ---------- ---------- ---------- ---------- ---------- ---------- ---------- ---------- ----------  1287

enFeLV-AGTT          1603 AAGGGAAGGC CCAAACAACA GACCCCAGTA CTGGCCATTC TCGGCCTCTG ACCTGTACAA TTGGAAATTG CATAACCCCC CTTTCTCCCA  1692
enFeLV-clone1        1591 .......... .......... .......... .......... .......... .......... .......... .......... ..........  1680
ChrB1:204342744      1557 .......... ..T....... .......... .......... .......... .......... .......... .......... ..........  1646
ChrB4:45570261       1566 .......... ..T....... .......... .......... .......... .......... .......... .......... ..........  1655
enFeLV-clone4        1287 ---------- ---------- ---------- ---------- ---------- ---------- ---------- ---------- ----------  1287

enFeLV-AGTT          1693 GGACCCAGTG GCCCTAACTA ACCTAATTGA GTCCATTTTA GTGACACATC AGCCAACCTG GGACGACTGC CAACAGCTCT TACAGGCTCT  1782
enFeLV-clone1        1681 .......... .......... .......... .......... .......... .......... .......... .......... ..........  1770
ChrB1:204342744      1647 .......... .......... .......... .......... .....G.... .......... ......T... .......... .....A....  1736
ChrB4:45570261       1656 .......... .......... .......... .......... .....G.... .......... ......T... .......... .....A....  1745
enFeLV-clone4        1287 ---------- ---------- ---------- ---------- ---------- ---------- ---------- ---------- ----------  1287

enFeLV-AGTT          1783 CCTGACGGCA GAGGAGAGAC AAAGGGTCCT CCTTGAAGCC CGAAAGCAAG TTCCAGGCGA GGACGGACGG CCAACCCAGC TGCCCAATGT  1872
enFeLV-clone1        1771 .......... .......... .......... .......... .......... .......... .......... .......... ..........  1860
ChrB1:204342744      1737 .......... .......... .......... .......... .......... ....G..... .......... .......... ..........  1826
ChrB4:45570261       1746 .......... .......... ...-...... .......... .......... ....G..... .......... .......... ..........  1834
enFeLV-clone4        1287 ---------- ---------- ---------- ---------- ---------- ---------- ---------- ---------- ----------  1287

enFeLV-AGTT          1873 CGTTGACGAG GCTTTCCCCT TGACCCGTCC CAACTGGGAT TTTGCTACGC CGGCAGGTAG GGAGCACCTA CGCCTTTATC GCCAGTTGCT  1962
enFeLV-clone1        1861 .......... .......... .......... .......... .......... .......... .......... .......... ..........  1950
ChrB1:204342744      1827 .......... .......... .......... .......... .......... .......... .......... .......... ..........  1916
ChrB4:45570261       1835 .......... .......... ......A... .......... .......... .......... ...------- -......... ..........  1916
enFeLV-clone4        1287 ---------- ---------- ---------- ---------- ---------- ---------- ---------- ---------- ----------  1287

enFeLV-AGTT          1963 GTTAGCGGGT CTCCGCGGGG CTGCAAGACG CCCCACTAAT TTGGCACAGG TAAAGCAAGT TGTACAAGGG AAAGAGGAAA CGCCAGCCTC  2052
enFeLV-clone1        1951 .......... .......... .......... .......... .......... .......... .......... .......... ..........  2040
ChrB1:204342744      1917 .......... .......... .......... .......... .......... .......... .......... .......... ........G.  2006
ChrB4:45570261       1917 .......... .......... .......... .......... .......... .......... .......... .......... ........G.  2006
enFeLV-clone4        1287 ---------- ---------- ---------- ---------- ---------- ---------- ---------- ---------- ----------  1287

enFeLV-AGTT          2053 ATTCTTAGAA AGATTAAAAG AGGCTTACAG AATGTACACT CCCTATGACC CTGAGGACCC AGGGCAGGCT GCTAGTGTTA TCCTGTCCTT  2142
enFeLV-clone1        2041 .......A.. .......... ..A....... ......T... .......... .......... .......... .......... ..........  2130
ChrB1:204342744      2007 .......... .......... .......... ......T... .......... .......T.. .......... .......... ..........  2096
ChrB4:45570261       2007 .......... .......... .......... ......T... .......... .......T.. .......... .......... ..........  2096
enFeLV-clone4        1287 ---------- ---------- ---------- ---------- ---------- ---------- ---------- ---------- ----------  1287
```

Figure S15-4

```
enFeLV-AGTT        2143 TATCTACCAG TCTAGCCCGG ACATAAGAAA TAAGTTACAA AGGCTAGAAG GCCTACAGGG GTTCACACTG TCTGATTTGC TAAAAGAGGC  2232
enFeLV-clone1      2131 .......... .......... .......... .......... .......... .......... .......... .......... ..........  2220
ChrB1:204342744    2097 .......... .......... .......... .......... ...T...... .......... .......... .......... ..........  2186
ChrB4:45570261     2097 .......... .......... .......... .......... ...T...... .......... .......... .......... ..........  2186
enFeLV-clone4      1287 ---------- ---------- ---------- ---------- ---------- ---------- ---------- ---------- ----------  1287

enFeLV-AGTT        2233 AGAAAAGATA TACAACAAAA GGGAGACCCC AGAGGAAAGG GAAGAAAGAT TATGGCAGCG GCAGGAAGAA AGAGATAAAA AGCGCCATAA  2322
enFeLV-clone1      2221 .......... .......... .......... .......... ...A...... .......... .......... ...A...... ..........  2310
ChrB1:204342744    2187 .......... .......... ....A..... ......G... .......... .......A.. .......... .......G.. ..........  2276
ChrB4:45570261     2187 .......... .......... ....A..... ......G... .......... .......A.. .......... .......... ..........  2276
enFeLV-clone4      1287 ---------- ---------- ---------- ---------- ---------- ---------- ---------- ---------- ----------  1287

enFeLV-AGTT        2323 GGAGATGACT AAAGTTCTGG CCACAGTAGT TGCTCAGAAT AGAGATAAGG ATAGAGAGGA AAGTAAACTG GGAGATCAAA GGAAAATACC  2412
enFeLV-clone1      2311 .......... .......... .......... .......... .......... .......... .......... .......... ..........  2400
ChrB1:204342744    2277 .......... .....C.... .......... .......... .......... ....G..A.. .G...G..A. .......G.. ..........  2366
ChrB4:45570261     2277 .......... .....C.... .......... .......... .......... ....G..A.. .G...G..A. .......G.. ..........  2366
enFeLV-clone4      1287 ---------- ---------- ---------- ---------- ---------- ---------- ---------- ---------- ----------  1287

enFeLV-AGTT        2413 TCTGGGGAAA GACCAGTGTG CCTATTGCAA GGAAAAGGGA CATTGGGTTC GCGATTGCCC GAAACGACCC CGGAAGAAAC CCGCAGACTC  2502
enFeLV-clone1      2401 .......... .......... .......... .......... .......... .......... .......... .......... ..........  2490
ChrB1:204342744    2367 .......... .......... .......... .......... .......... .......... .......... .......... .......---  2453
ChrB4:45570261     2367 .......... .......... .......... .......... .......... .......... .......... .......... .......---  2453
enFeLV-clone4      1287 ---------- ---------- ---------- ---------- ---------- ---------- ---------- ---------- ----------  1287

enFeLV-AGTT        2503 CACTCTCCTC AACCTAGAAG ATTAGGAGAG TCAGGGCCAG GA-CCCCCCC CCTGAGCCCA GGATAACCCT AAAAGTAGGG GGGCAACCAG  2591
enFeLV-clone1      2491 .......... .......... .......... .......... ..C....... .......... .......... .......... ..........  2580
ChrB1:204342744    2453 ---------- ---------- ---------- ---------- ---------- ---------- ---------- ---------- ----------  2453
ChrB4:45570261     2453 ---------- ---------- ---------- ---------- ---------- ---------- ---------- ---------- ----------  2453
enFeLV-clone4      1287 ---------- ---------- ---------- ---------- ---------- ---------- ---------- ---------- ----------  1287

enFeLV-AGTT        2592 TGACCTTTCT GGTGGATACG GGAGCCCAGC ACTCGGTACT GACTCGACCA GATGGACCTC TCAGTGACCG CTCAGCCCTG GTGCAAGGAG  2681
enFeLV-clone1      2581 .......... .......... .......... .......... .......... .......... .......... .......... ..........  2670
ChrB1:204342744    2453 ---------- ---------- ---------- ---------- ---------- ---------- ---------- ---------- ----------  2453
ChrB4:45570261     2453 ---------- ---------- ---------- ---------- ---------- ---------- ---------- ---------- ----------  2453
enFeLV-clone4      1287 ---------- ---------- ---------- ---------- ---------- ---------- ---------- ---------- ----------  1287

enFeLV-AGTT        2682 CCACAGGAAG CAAAAACTAC CGATGGACCA CCGACAGGAG GGTGCAACTG GCAACCGGTA AGGTGACTCA TTCTTTTTTA TATGTACCTG  2771
enFeLV-clone1      2671 .......... .......... .......... .......... .......... .......... .......... .......... ..........  2760
ChrB1:204342744    2453 ---------- ---------- ---------- ---------- ---------- ---------- ---------- ---------- ----------  2453
ChrB4:45570261     2453 ---------- ---------- ---------- ---------- ---------- ---------- ---------- ---------- ----------  2453
enFeLV-clone4      1287 ---------- ---------- ---------- ---------- ---------- ---------- ---------- ---------- ----------  1287

enFeLV-AGTT        2772 AATGTCCCTA CCCGTTATTA GGGAGAGACC TATTAACCAA ACTCAAGGCC CAGATCCATT TCACCGGAGA AGGAGCTAAT GTTGTTGGAC  2861
enFeLV-clone1      2761 .......... .......... .......... .......... .......... .......... .......... .......... ..........  2850
ChrB1:204342744    2453 ---------- ---------- ---------- ---------- ---------- ---------- ---------- ---------- ----------  2453
ChrB4:45570261     2453 ---------- ---------- ---------- ---------- ---------- ---------- ---------- ---------- ----------  2453
enFeLV-clone4      1287 ---------- ---------- ---------- ---------- ---------- ---------- ---------- ---------- ----------  1287
```

Figure S15-5

```
enFeLV-AGTT          2862 CCATGGGCTT ACCCCTACAA GTCCTCACCT TGCAACTAGA AGAAGAGTAT CGGCTATTTG AGCCGGAAAG TGAACTAAAA CAAGGTATGG  2951
enFeLV-clone1        2851 .......... .......... .......... .......... .......... .......... .......... .......... ..........  2940
ChrB1:204342744      2453 ---------- ---------- ---------- ---------- ---------- ---------- ---------- ---------- ----------  2453
ChrB4:45570261       2453 ---------- ---------- ---------- ---------- ---------- ---------- ---------- ---------- ----------  2453
enFeLV-clone4        1287 ---------- ---------- ---------- ---------- ---------- ---------- ---------- ---------- ----------  1287

enFeLV-AGTT          2952 ACAGTTGGCT TAAAAACTTT CCCCAGGCAT GGGCAGAAAC AGGAGGTATA GGAATGGCTC ATTGCCAAGC CCCCATCCTC ATTCAACTTA  3041
enFeLV-clone1        2941 .......... .......... .......... .......... .......... .......... .......... .......... ..........  3030
ChrB1:204342744      2453 ---------- ---------- ---------- ---------- ---------- ---------- ---------- ---------- ----------  2453
ChrB4:45570261       2453 ---------- ---------- ---------- ---------- ---------- ---------- ---------- ---------- ----------  2453
enFeLV-clone4        1287 ---------- ---------- ---------- ---------- ---------- ---------- ---------- ---------- ----------  1287

enFeLV-AGTT          3042 AAGCTACTGC CACCCCAATC TCCATCCGGC AGTACCCCAT GCCCCATGAA GCTTACCAAG GAATTAAACC CCATATAAGG AGAATGCTGG  3131
enFeLV-clone1        3031 .......... .......... .......... .......... .......... .......... .......... .......... ..........  3120
ChrB1:204342744      2453 ---------- ---------- ---------- ---------- ---------- ---------- ---------- ---------- ----------  2453
ChrB4:45570261       2453 ---------- ---------- ---------- ---------- ---------- ---------- ---------- ---------- ----------  2453
enFeLV-clone4        1287 ---------- ---------- ---------- ---------- ---------- ---------- ---------- ---------- ----------  1287

enFeLV-AGTT          3132 ACCAAGGCAT CCTCAAGCCC TGCCAGTCCC CATGGAATAC ACCCCTATTA CCTGTCAAAA AGCCAGGAAC CGGGGATTAC AGACCAGTGC  3221
enFeLV-clone1        3121 .......... .......... .......... .......... .......... .......... .......... .......... ..........  3210
ChrB1:204342744      2453 ---------- ---------- ---------- ---------- ---------- ---------- ---------- ---------- ----------  2453
ChrB4:45570261       2453 ---------- ---------- ---------- ---------- ---------- ---------- ---------- ---------- ----------  2453
enFeLV-clone4        1287 ---------- ---------- ---------- ---------- ---------- ---------- ---------- ---------- ----------  1287

enFeLV-AGTT          3222 AGGACTTAAG AGAAGTAAAC AAAAGGGTAG AAGACATCCA TCCCACTGTG CCAAACCCAT ACAACCTCCT TAGCACCCTC CCACCTTCTC  3311
enFeLV-clone1        3211 .......... .......... .......... .......... .......... .......... .......... .......... ..........  3300
ChrB1:204342744      2453 ---------- ---------- ---------- ---------- ---------- ---------- ---------- ---------- ----------  2453
ChrB4:45570261       2453 ---------- ---------- ---------- ---------- ---------- ---------- ---------- ---------- ----------  2453
enFeLV-clone4        1287 ---------- ---------- ---------- ---------- ---------- ---------- ---------- ---------- ----------  1287

enFeLV-AGTT          3312 ACCCTTGGTA CACCGTCCTA GATCTAAAAG ATGCTTTTTT CTGCTTGCGA CTACACCCTG AGAGCCAATT ACTCTTTGCA TTTGAATGGA  3401
enFeLV-clone1        3301 .......... .......... ....C..... .......... .......... .......... .......... .......... ..........  3390
ChrB1:204342744      2453 ---------- ---------- ---------- ---------- ---------- ---------- ---------- ---------- ----------  2453
ChrB4:45570261       2453 ---------- ---------- ---------- ---------- ---------- ---------- ---------- ---------- ----------  2453
enFeLV-clone4        1287 ---------- ---------- ---------- ---------- ---------- ---------- ---------- ---------- ----------  1287

enFeLV-AGTT          3402 AAGATCCAGA GATAGGGCTG TCAGGGCAAC TGACCTGGAC TCGCCTTCCA CAAGGGTTCA AGAATAGCCC CACCCTATTT GATGAGGCCC  3491
enFeLV-clone1        3391 .......... .......... .......... .......... .......... .......... .......... .......... ..C.......  3480
ChrB1:204342744      2453 ---------- ---------- ---------- ---------- ---------- ---------- ---------- ---------- ----------  2453
ChrB4:45570261       2453 ---------- ---------- ---------- ---------- ---------- ---------- ---------- ---------- ----------  2453
enFeLV-clone4        1287 ---------- ---------- ---------- ---------- ---------- ---------- ---------- ---------- ----------  1287

enFeLV-AGTT          3492 TACACTCAGA CCTGGCAGAT TTCAGGGTGA GGTATCCAGC TTTAGTCCTC CTACAATACG TAGATGACCT CTTACTGGCT GCGGCAACCA  3581
enFeLV-clone1        3481 .......... .......... .......... .......... .......... ........T. .......... .......... ..........  3570
ChrB1:204342744      2453 ---------- ---------- ---------- ---------- ---------- ---------- ---------- ---------- ----------  2453
ChrB4:45570261       2453 ---------- ---------- ---------- ---------- ---------- ---------- ---------- ---------- ----------  2453
enFeLV-clone4        1287 ---------- ---------- ---------- ---------- ---------- ---------- ---------- ---------- ----------  1287
```

Figure S15-6

```
enFeLV-AGTT        3582 AGACCGAATG CCTAGAAGGG ACTAAAGCAC TCCTTGAGAC TTTGGGCAAT AAGGGTTACC GAGCCTCTGC AAAGAAGGCC CAAATTTGCC  3671
enFeLV-clone1      3571 .......... .......... .....G.... .......... .......... .......... .......... .......... ..........  3660
ChrB1:204342744    2453 ---------- ---------- ---------- ---------- ---------- ---------- ---------- ---------- ----------  2453
ChrB4:45570261     2453 ---------- ---------- ---------- ---------- ---------- ---------- ---------- ---------- ----------  2453
enFeLV-clone4      1287 ---------- ---------- ---------- ---------- ---------- ---------- ---------- ---------- ----------  1287

enFeLV-AGTT        3672 TGCAAAAAGT CACATACCTA GGGTACTCTT TAGAGGATGG TCAAAGATGG CTTACCAAAG CTCGGAAAGA AGCCATTCTC TCCATCCCTG  3761
enFeLV-clone1      3661 .......... .......... .......... .......... .......... .......... .......... .......... ..........  3750
ChrB1:204342744    2453 ---------- ---------- ---------- ---------- ---------- ---------- ---------- ---------- ----------  2453
ChrB4:45570261     2453 ---------- ---------- ---------- ---------- ---------- ---------- ---------- ---------- ----------  2453
enFeLV-clone4      1287 ---------- ---------- ---------- ---------- ---------- ---------- ---------- ---------- ----------  1287

enFeLV-AGTT        3762 TGCCTAAAAA CCCACGGCAA GTGAGAGAGT TCCTTGGAAC TGCAGGCTAC TGCCGGCTGT GGATTCCCGG TTTTGCCGAG CTCGCTGCCC  3851
enFeLV-clone1      3751 .......... .......... .......... .......... .......... .......... .......... .......... ..........  3840
ChrB1:204342744    2453 ---------- ---------- ---------- ---------- ---------- ---------- ---------- ---------- ----------  2453
ChrB4:45570261     2453 ---------- ---------- ---------- ---------- ---------- ---------- ---------- ---------- ----------  2453
enFeLV-clone4      1287 ---------- ---------- ---------- ---------- ---------- ---------- ---------- ---------- ----------  1287

enFeLV-AGTT        3852 CGCTATATCC TCTCACTCGA CCAGGAACTC TGTTTCAGTG GGGAACAGAA CAACAATTGG CCTTCGAAAA CATTAAAAAA GCCCTCTTGA  3941
enFeLV-clone1      3841 .......... .......... .......... .......... .......A.. .......... .......... .......... ..........  3930
ChrB1:204342744    2453 ---------- ---------- ---------- ---------- ---------- ---------- ---------- ---------- ----------  2453
ChrB4:45570261     2453 ---------- ---------- ---------- ---------- ---------- ---------- ---------- ---------- ----------  2453
enFeLV-clone4      1287 ---------- ---------- ---------- ---------- ---------- ---------- ---------- ---------- ----------  1287

enFeLV-AGTT        3942 GCTCCCCTGC CCTGGGGTTG CCAGATATCA CCAAGCCCTT TGAATTATTT ATTGATGAGA GCTCAGGGTT TGCGAAGGGG GTGTTAGTCC  4031
enFeLV-clone1      3931 .......... .......... .......... .......... .......... .......... .......... .......... ..........  4020
ChrB1:204342744    2453 ---------- ---------- ---------- ---------- ---------- ---------- ---------- ---------- ----------  2453
ChrB4:45570261     2453 ---------- ---------- ---------- ---------- ---------- ---------- ---------- ---------- ----------  2453
enFeLV-clone4      1287 ---------- ---------- ---------- ---------- ---------- ---------- ---------- ---------- ----------  1287

enFeLV-AGTT        4032 AAAAACTGGG ACCCTGGAAA AGACCAGTTG CCTACCTATC AAAAAAACTG GATACAGTGG CATCTGGATG GCCCCCTTGT TTACGCATGG  4121
enFeLV-clone1      4021 .......... .......... .......... .......... .......... .......... .......... .......... ..........  4110
ChrB1:204342744    2453 ---------- ---------- ---------- ---------- ---------- ---------- ---------- ---------- ----------  2453
ChrB4:45570261     2453 ---------- ---------- ---------- ---------- ---------- ---------- ---------- ---------- ----------  2453
enFeLV-clone4      1287 ---------- ---------- ---------- ---------- ---------- ---------- ---------- ---------- ----------  1287

enFeLV-AGTT        4122 TTGCAGCCAT CGCCATCCTA GTCAAGGATG CAGGGAAGCT AACTCTAGGA CAGCCGCTAA CTGTCCTGAC CTCCCACCCA GTTGAGGCAC  4211
enFeLV-clone1      4111 .......... .......... .......... .......... .......... .......... .......... .......... ..........  4200
ChrB1:204342744    2453 ---------- ---------- ---------- ---------- ---------- ---------- ---------- ---------- ----------  2453
ChrB4:45570261     2453 ---------- ---------- ---------- ---------- ---------- ---------- ---------- ---------- ----------  2453
enFeLV-clone4      1287 ---------- ---------- ---------- ---------- ---------- ---------- ---------- ---------- ----------  1287

enFeLV-AGTT        4212 TTGTCCGACA GCCTCCAAAT AAATGGCTCT CTAATGCTAG AATGACTCAT TACCAAGCTA TGCTCCTCGA TGCAGAGCGA GTCCATTTCG  4301
enFeLV-clone1      4201 .......... .......... .......... .......... .......... .......... .......... .......... ..........  4290
ChrB1:204342744    2453 ---------- ---------- ---------- ---------- ---------- ---------- ---------- ---------- ----------  2453
ChrB4:45570261     2453 ---------- ---------- ---------- ---------- ---------- ---------- ---------- ---------- ----------  2453
enFeLV-clone4      1287 ---------- ---------- ---------- ---------- ---------- ---------- ---------- ---------- ----------  1287
```

Figure S15-7

```
enFeLV-AGTT          4302 GGCCAACAGT CTCCCTTAAC CCTGCCACCT TGCTTCCCCT CCCCAACGAG GGAAACCACC ACGACTGCCT CCAGATTTTA GCCGAGACCC  4391
enFeLV-clone1        4291 .......... .......... .......... .......... .......... .......... .......... .......... ..........  4380
ChrB1:204342744      2453 ---------- ---------- ---------- ---------- ---------- ---------- ---------- ---------- ----------  2453
ChrB4:45570261       2453 ---------- ---------- ---------- ---------- ---------- ---------- ---------- ---------- ----------  2453
enFeLV-clone4        1287 ---------- ---------- ---------- ---------- ---------- ---------- ---------- ---------- ----------  1287

enFeLV-AGTT          4392 ATGGCACCAG ACCCGACTTA ACTGACCAGC CGTTGCCGGA TGCAGACCTG ACCTGGTACA CGGATGGTAG CAGCTTCATC CGTAACGGAG  4481
enFeLV-clone1        4381 .......... .......... .......... .......... .......... .......... .......... .......... ..........  4470
ChrB1:204342744      2453 ---------- ---------- ---------- ---------- ---------- ---------- ---------- ---------- ----------  2453
ChrB4:45570261       2453 ---------- ---------- ---------- ---------- ---------- ---------- ---------- ---------- ----------  2453
enFeLV-clone4        1287 ---------- ---------- ---------- ---------- ---------- ---------- ---------- ---------- ----------  1287

enFeLV-AGTT          4482 AGAGAAAGGC CGGAGCCGCA GTAACAACCG AATCTGAGGT AATCTGGGCT GCTTCCCTCC CACCCGGAAC GTCAGCCCAG CGAGCCGAAC  4571
enFeLV-clone1        4471 .......... .......... .......... .......... .......... .......... .......... .......... ..........  4560
ChrB1:204342744      2453 ---------- ---------- ---------- ---------- ---------- ---------- ---------- ---------- ----------  2453
ChrB4:45570261       2453 ---------- ---------- ---------- ---------- ---------- ---------- ---------- ---------- ----------  2453
enFeLV-clone4        1287 ---------- ---------- ---------- ---------- ---------- ---------- ---------- ---------- ----------  1287

enFeLV-AGTT          4572 TGATTGCCCT GACCCAGGCA CTGAAGATGG CAAAAGGTAA GAAGCTAACT GTCTATACGG ACAGCCGATA TGCCTTTGCT ACAGCTCATG  4661
enFeLV-clone1        4561 .......... .......... .......... .......... .......... .......... .......... .......... ..........  4650
ChrB1:204342744      2453 ---------- ---------- ---------- ---------- ---------- ---------- ---------- ---------- ----------  2453
ChrB4:45570261       2453 ---------- ---------- ---------- ---------- ---------- ---------- ---------- ---------- ----------  2453
enFeLV-clone4        1287 ---------- ---------- ---------- ---------- ---------- ---------- ---------- ---------- ----------  1287

enFeLV-AGTT          4662 TACACGGGGA AATCTACAGG CGGCGGGGCC TGCTAACTTC AGAAGGAAAA GAAATTAAAA ATAAAAATGA AATCCTCGCC CTATTAGAGG  4751
enFeLV-clone1        4651 .......... .......... .......... .......... .......... .......... .......... .......... ..........  4740
ChrB1:204342744      2453 ---------- ---------- ---------- ---------- ---------- ---------- ---------- ---------- ----------  2453
ChrB4:45570261       2453 ---------- ---------- ---------- ---------- ---------- ---------- ---------- ---------- ----------  2453
enFeLV-clone4        1287 ---------- ---------- ---------- ---------- ---------- ---------- ---------- ---------- ----------  1287

enFeLV-AGTT          4752 CGTTATTCTT ACCCAAAAGA CTGAGTATCA TCCATTGCCC TGGACACCAA AAAGGTGATA GTCCCCAGGC GAAAGGAAAC AGATTAGCCG  4841
enFeLV-clone1        4741 .......... .......... .......... .......... G......... .......... .......... .......... ..........  4830
ChrB1:204342744      2453 ---------- ---------- ---------- ---------- ---------- ---------- ---------- ---------- ----------  2453
ChrB4:45570261       2453 ---------- ---------- ---------- ---------- ---------- ---------- ---------- ---------- ----------  2453
enFeLV-clone4        1287 ---------- ---------- ---------- ---------- ---------- ---------- ---------- ---------- ----------  1287

enFeLV-AGTT          4842 ATGATACAGC AAAGAAAGCC GCCACAGAGA CTCAATCATC ACTAACCATC TTACCCACTG AACTTATAGA GGGTCCCAAA AGGCCTCCAT  4931
enFeLV-clone1        4831 .......... .......... .......... .......... .......... .......... .......... .......... ..........  4920
ChrB1:204342744      2453 ---------- ---------- ---------- ---------- ---------- ---------- ---------- ---------- ----------  2453
ChrB4:45570261       2453 ---------- ---------- ---------- ---------- ---------- ---------- ---------- ---------- ----------  2453
enFeLV-clone4        1287 ---------- ---------- ---------- ---------- ---------- ---------- ---------- ---------- ----------  1287

enFeLV-AGTT          4932 GGGAATATGA TGACAGTGAT TTAGACCTTG TGCAGAAACT TGAAGCTCAT TATGAGCCAA AAAGAGGTAC CTGGGAGTAC CAAGGGAAAA  5021
enFeLV-clone1        4921 .......... .......... .......... .......... .......... .......... .......... .......... ..........  5010
ChrB1:204342744      2453 ---------- ---------- ---------- ---------- ---------- ---------- ---------- ---------- ----------  2453
ChrB4:45570261       2453 ---------- ---------- ---------- ---------- ---------- ---------- ---------- ---------- ----------  2453
enFeLV-clone4        1287 ---------- ---------- ---------- ---------- ---------- ---------- ---------- ---------- ----------  1287
```

Figure S15-8

```
enFeLV-AGTT        5022 CTATAATGCC TGAAAAATAC GCAAAAGAGT TGATTAGCCA TCTGCATAAG TTAACACACC TCAGTGCTAG GAAAATGAAA ACTTTACTAG  5111
enFeLV-clone1      5011 .......... .......... .......... .......... .......... .......... .......... .......... ..........  5100
ChrB1:204342744    2453 ---------- ---------- ---------- ---------- ---------- ---------- ---------- ---------- ----------  2453
ChrB4:45570261     2453 ---------- ---------- ---------- ---------- ---------- ---------- ---------- ---------- ----------  2453
enFeLV-clone4      1287 ---------- ---------- ---------- ---------- ---------- ---------- ---------- ---------- ----------  1287

enFeLV-AGTT        5112 AAAGAGAAGA AACTGGGTTT TACCTCCCTA ACAGAGACTT ACACCTCCGG CAAGTAACAG AGAGCTGCCG GGCATGTGCT CAAATCAACG  5201
enFeLV-clone1      5101 .......... .......... .......... .......... .......... .......... .......... .......... ..........  5190
ChrB1:204342744    2453 ---------- ---------- ---------- ---------- ---------- ---------- ---------- ---------- ----------  2453
ChrB4:45570261     2453 ---------- ---------- ---------- ---------- ---------- ---------- ---------- ---------- ----------  2453
enFeLV-clone4      1287 ---------- ---------- ---------- ---------- ---------- ---------- ---------- ---------- ----------  1287

enFeLV-AGTT        5202 CAGGGAAAAT AAAGTTTGGA CCTGATGTAA GGGCCCGAGG CCACCGGCCC GGAATACATT GGGAAGTAGA CTTCACTGAA ATCAAGCCAG  5291
enFeLV-clone1      5191 .......... .......... .......... .......... .......... .......... .......... .......... ..........  5280
ChrB1:204342744    2453 ---------- ---------- ---------- ---------- ---------- ---------- ---------- ---------- ----------  2453
ChrB4:45570261     2453 ---------- ---------- ---------- ---------- ---------- ---------- ---------- ---------- ----------  2453
enFeLV-clone4      1287 ---------- ---------- ---------- ---------- ---------- ---------- ---------- ---------- ----------  1287

enFeLV-AGTT        5292 GAATGTATGG ATATAAATAC CTCTTGGTGT TCATAGACAC CTTCTCTGGC TGGGCCGAAG CTTACCCCGC CAAACATGAA ACAGCAAAAG  5381
enFeLV-clone1      5281 .......... .......... .......... .......... .......... .......... .......... .......... ..........  5370
ChrB1:204342744    2453 ---------- ---------- ---------- ---------- ---------- ---------- ---------- ---------- ----------  2453
ChrB4:45570261     2453 ---------- ---------- ---------- ---------- ---------- ---------- ---------- ---------- ----------  2453
enFeLV-clone4      1287 ---------- ---------- ---------- ---------- ---------- ---------- ---------- ---------- ----------  1287

enFeLV-AGTT        5382 TTGTTGCCAA GAAACTCTTA GAAGAGATTT TTCCTCGTTA CGGGATCCCT CAGGTATTGG GTTCAGATAA TGGACCCGCC TTTATCTCCC  5471
enFeLV-clone1      5371 .......... .......... .......... .......... .......... .......... .......... .......... ..........  5460
ChrB1:204342744    2453 ---------- ---------- -----..... .......... .......... .......... .......... .......... ..C.......  2518
ChrB4:45570261     2453 ---------- ---------- -----..... .......... .......... .......... .......... .......... ..C.......  2518
enFeLV-clone4      1287 ---------- ---------- ---------- ---------- ---------- ---------- ---------- ..CC...T.. .AAGCA....  1307

enFeLV-AGTT        5472 AGGTAAGTCA GTCTGTGGCC ACCCTACTGG GGATTAATTG GAAATTACAT TGTGCATACC GACCCCAAAG TTCAGGTCAG GTAGAAAGAA  5561
enFeLV-clone1      5461 .......... .......... .......... .......... .......... .......... .......... .......... ..........  5550
ChrB1:204342744    2519 .......... .......... .......... .......... .......... .......... ......T... .......... ..........  2608
ChrB4:45570261     2519 .......... .......... .......... .......... .......... .......... .......... .......... ..........  2608
enFeLV-clone4      1308 .......... .......... .......... .......... .......... .......... .......... .......... ..........  1397

enFeLV-AGTT        5562 TGAATAGATC AATTAAGGAG ACTTTAACTA AATTAACGCT AGAAACTGGC TCTAAGGATT GGGTGCTCCT CCTGCCCCTG GTTTTATACC  5651
enFeLV-clone1      5551 .......... .......... .......... .......... .......... .......... .......... .......... ..........  5640
ChrB1:204342744    2609 .......... .......... .......... .......... .......... .......... .......... .......... ..........  2698
ChrB4:45570261     2609 .......... .......... .......... .......... .......... .......... .......... .......... ..........  2698
enFeLV-clone4      1398 .......... .......... .......... .......... .......... .......... .......... .......... ..........  1487

enFeLV-AGTT        5652 GGGTACGTAA CACGCCAGGT CCCCACGGGT TAACTCCTTT TGAAATCCTG TACGGGGCAC CCCCACCTAT GGCTCACTTC TTTGATGCTG  5741
enFeLV-clone1      5641 .......... .......... .......... .......... .......... .....A.... .......... .......... ..........  5730
ChrB1:204342744    2699 .......... .......... .......... .......... .......... .......... .T........ .......... ......T...  2788
ChrB4:45570261     2699 .......... .......... ........A. .......... .......... ...A...... .T........ .......... ......T...  2788
enFeLV-clone4      1488 .......... .......... .......... .......... .......... .......... .......... .......... ..........  1577
```

Figure S15-9

```
                                   env
enFeLV-AGTT       5742 ACATCTCTGG TTTCGCTACC TCCCCCACCA TGCAGGCACA TTTACGCGCC CTGCAGCTGG TCCAAGAAGA GATCCAGAGA CCTCTAGCGG  5831
enFeLV-clone1     5731 .......... .......... .......... .......... .......... .......... .......... .......... ..........  5820
ChrB1:204342744   2789 .......... .........A .......... .......... .......... .......... .......... A......... ..........  2878
ChrB4:45570261    2789 .......... .........A .......... .......... .......... .......... .......... A......... ..........  2878
enFeLV-clone4     1578 .......... .......... .......... .......... .......... .......... .......... .......... ..........  1667

enFeLV-AGTT       5832 CAGCCTACCG AGAAAAGCTC GAAACCCCGG TTGTGCCTCA CCCCTTCAAA CCAGGAGACT CCGTCTGGGT TCGGAGACAT CAAACCAAGA  5921
enFeLV-clone1     5821 .......... .......... .......... .......... .......... .......... .......... .......... ..........  5910
ChrB1:204342744   2879 .......... .......... .......... .......... .......... .......... .......... .......... ..........  2968
ChrB4:45570261    2879 .......... .......... .......... .......... .......... .......... .......... .......... ..........  2968
enFeLV-clone4     1668 .......... .......... .......T.. .......... .......... .......... ..A....... .......... ..........  1757

enFeLV-AGTT       5922 ACCTCGAGCC ACGGTGGAAG GGACCACATA TCGTCCTCCT GACCACCCCC ACGGCCTTAA AAGTAGACGG AGTTGCTGCT TGGATCCACG  6011
enFeLV-clone1     5911 .......... .......... .......... .......... .......... .......... .......... .......... ..........  6000
ChrB1:204342744   2969 .......... .......... .......... .......... .......... .......... .......... .......... ..........  3058
ChrB4:45570261    2969 .......... .......... .......... .......... .......... .......... .......... .......... ..........  3058
enFeLV-clone4     1758 .......... .......... .......... .......... .......... .......... .......... .......... ..........  1847

                                    env
enFeLV-AGTT       6012 CCTCACATGT AAAGGCTGCA GGGCCAACCA CCAATCAGGA CCCCTCAGAC GACCCCAGCT CAGACGATCC ATCGAGATGG AAGGTCCAAC  6101
enFeLV-clone1     6001 .......... .......... .......... .......... .......... .......... .......... .......... ..........  6090
ChrB1:204342744   3059 ....T..... .......... .......... .......... .......... .......... .........T ...A...... ..........  3148
ChrB4:45570261    3059 ....T..... .......... .......... .......... .......... .......... ......G..T ...A...... ..........  3148
enFeLV-clone4     1848 .......... .......... .......... .......... .......... .......... .......... .......... ..........  1937

                                                         gag-pol
enFeLV-AGTT       6102 GCACCCAAAA CCCTCTAAAG ATAAGACTTT CTCGTGGGAC CTAATGATTC TGGTGGGGGT CTTATTAAGA CTAGACGTGG GAATGGCCAA  6191
enFeLV-clone1     6091 .......... .......... .......... .......... .......... .......... .......... .......... ..........  6180
ChrB1:204342744   3149 .......... .......... .......... .......... .....A.... .......... ....C..... .......... ..........  3238
ChrB4:45570261    3149 .......... .......... .......... .......... .....A.... .......... ....C..... .......... ..........  3238
enFeLV-clone4     1938 .......... .......... .......... .......... .......... .......... .......... .......... ..........  2027

enFeLV-AGTT       6192 TCCTAGTCCG CACCAAGTGT ATAATGTAAC TTGGACAATA ACCAACCTTG TAACTGGAAC AAAGGCTAAT GCCACCTCCA TGTTGGGAAC  6281
enFeLV-clone1     6181 .......... .......... .......... .......... .......... .......... .......... .......... ..........  6270
ChrB1:204342744   3239 .......... .......... .....A.... .......... .......... .......... .......... .......... ..........  3328
ChrB4:45570261    3239 .......... .......... .....A.... .......... .......... .......... .......... .......... ..........  3328
enFeLV-clone4     2028 .......... .......... .......... .......... .......... .......... .......... .......... ..........  2117

enFeLV-AGTT       6282 CCTGACAGAC GCCTTCCCTA CCATGTATTT TGACTTATGT GATATAATAG GAAATACATG GAACCCTTCA GATCAGGAAC CATTCCCAGG  6371
enFeLV-clone1     6271 .......... .......... .......... .......... .......... .......... .......... .......... ..........  6360
ChrB1:204342744   3329 .......... .......... ..C.A..... .......... .......... .......... .......... .G........ ..........  3418
ChrB4:45570261    3329 .......... .......... ..C.A..... .......... .......... .......... .......... .G........ ..........  3418
enFeLV-clone4     2118 .......... .......... .......... .......... .......... .......... .......... .......... ..........  2207

enFeLV-AGTT       6372 GTATGGATGT GATCAGCCTA TGAGGAGGTG GCAACAGAGA AACACACCCT TTTATGTCTG TCCAGGACAT GCCAACCGGA AGCAATGTGG  6461
enFeLV-clone1     6361 .......... .......... .......... .......... .......... .......... .......... .......... ..........  6450
ChrB1:204342744   3419 ...C...... .......... .......... ...G...... ......G... .......... .......... .......... ..........  3508
ChrB4:45570261    3419 ...C...... .......... .......... ...G...... ......G... .......... .......... .......... ..........  3508
enFeLV-clone4     2208 .......... .......... .......... .......... .......... .......... .......... .......... ..........  2297
```

Figure S15-10

```
enFeLV-AGTT       6462 GGGGCCACAG GATGGGTTCT GCGCTGTATG GGGTTGCGAG ACCACCGGGG AAACCTATTG GAGACCCACC TCCTCATGGG ACTACATCAC  6551
enFeLV-clone1     6451 .......... .......... .......... .......... .......... .......... .......... .......... ..........  6540
ChrB1:204342744   3509 .......... .......... ....C..... .......... .......... .G........ ..A....... .....C.... ..........  3598
ChrB4:45570261    3509 .......... .......... ....C..... .......... .......... .G........ ..A....... .....C.... ..........  3598
enFeLV-clone4     2298 .......... .......... .......... .......... .......... .......... .......... .......... ..........  2387

enFeLV-AGTT       6552 AGTAAAAAAA GGGGTTACTC AGGGAATATA TCAATGTAGT GGAGGTGGTT GGTGTGGGCC CTGTTACGAT AAAGCTGTTC ACTCCTCGAA  6641
enFeLV-clone1     6541 .......... .......... .......... .......... .......... .......... .......... .......... .........T  6630
ChrB1:204342744   3599 .......... .......... .......... .......... .......... .......... .......... .......... .........C  3688
ChrB4:45570261    3599 .......... .......... .......... .......... .......... .......... .......... .......... .........C  3688
enFeLV-clone4     2388 .......... .......... .......... .......... .......... .......... .......... .......... .........T  2477

enFeLV-AGTT       6642 AACGGGAGCT AGTGAAGGGG GCCGGTGCAA CCCCTTGATC TTGCAATTTA CCCAAAAGGG AAGACAAACA TCTTGGGATG GACCTAAGTC  6731
enFeLV-clone1     6631 .......... .......... .......... .......... .......... .......... .......... .......... ..........  6720
ChrB1:204342744   3689 .......... .......... .......... .......... .......... .......... .........G .......... ..........  3778
ChrB4:45570261    3689 .......... .......... .......... .......... .......... .......... .......... .......... ..........  3778
enFeLV-clone4     2478 .......... .......... .......... .......... .......... .......... .......... .......... ..........  2567

enFeLV-AGTT       6732 ATGGGGGCTA CGACTATACC GTTCAGGATA TGACCCTATA GCCCTGTTCT CGGTATCCCG GCAAGTAATG ACCATTACGC CGCCTCAGGC  6821
enFeLV-clone1     6721 .......... .......... .......... .......... .......... .......... .......... .......... ..........  6810
ChrB1:204342744   3779 .......... .......... .......... .......... .......... .......... .......... .......... ..........  3868
ChrB4:45570261    3779 .......... .......... .......... ...T...... .......... .......... .......... .......... ..........  3868
enFeLV-clone4     2568 .......... .......... .......... .......... .......... .......... .......... .......... ..........  2657

enFeLV-AGTT       6822 CATGGGACCA AATCTAGTCC TGCCTGATCA AAAACCCCCA TCCAGGCAAT CTCAAATAGA GTCCCGAGTA ACACCTCACC ATTCCCAAGG  6911
enFeLV-clone1     6811 .......... .......... .......... .......... .......... .......... .......... .......... ..........  6900
ChrB1:204342744   3869 .......... G...C..... .......... .......... .......... .......... .......... .T.......- ----------  3947
ChrB4:45570261    3869 .......... ....C..... .......... .......... .......... .......... .......... .T.......- ----------  3947
enFeLV-clone4     2658 .......... .......... .......... .......... .......... .......... .......... .......... ..........  2747

enFeLV-AGTT       6912 CAACGGAGGC ACCCCAGGTA TAACTCTTGT TAATGCCTCC ATTGCCCCTC TAAGTACCCC TGTCACCCCC GCAAGTCCCA AACGTATAGG  7001
enFeLV-clone1     6901 .......... .......... .......... .......... .......... .......... .......... .......... ..........  6990
ChrB1:204342744   3947 ---------- ---------- ---------- ---------- ---------- ---------- ---------- ---------- ----------  3947
ChrB4:45570261    3947 ---------- ---------- ---------- ---------- ---------- ---------- ---------- ---------- ----------  3947
enFeLV-clone4     2748 .......... .......... .......... .......... .......... .......... .......... .......... ..........  2837

enFeLV-AGTT       7002 GACAGGAAAT AGGTTAATAA ATTTAGTGCA GGGGACATAT CTAGCTTTAA ATGTCACTAA CCCCAACAAA ACTAAAGACT GTTGGCTCTG  7091
enFeLV-clone1     6991 .......... .......... .......... .......... .......... .......... .......... .......... ..........  7080
ChrB1:204342744   3947 ---------- ---------- ---------- ---------- ---------- ---------- ---------- ---------- ----------  3947
ChrB4:45570261    3947 ---------- ---------- ---------- ---------- ---------- ---------- ---------- ---------- ----------  3947
enFeLV-clone4     2838 .......... .......... .......... .......... .......... .......... .......... .......... ..........  2927

enFeLV-AGTT       7092 TCTAGTCTCC CGACCGCCCT ATTATGAAGG AATTGCGGTA TTGGGCAATT ACAGCAACCA AACCAA---- CCCCCCCCCA TCCTGCCTAT  7177
enFeLV-clone1     7081 .......... .......... .........A .......... .......... .......... ......---- .......... ..........  7166
ChrB1:204342744   3947 ---------- ---------- ---------- ---------- ---------- ---------- ---------- ---------- ----------  3947
ChrB4:45570261    3947 ---------- ---------- ---------- ---------- ---------- ---------- ---------- ---------- ----------  3947
enFeLV-clone4     2928 .......... .......... .C........ .......... .......... .......... ......CCCC .......... ..........  3017
```

Figure S15-11

```
enFeLV-AGTT        7178 CCGACCCACA ACATAAACTG ACTATATCAG AAGTGTCCGG GCAAGGTTTG TGCATAGGGA CTGTTCCTAA GACCCACCAA GCTTTGTGCA  7267
enFeLV-clone1      7167 .......... .......... .......... .......... .......... .......... .......... .......... ..........  7256
ChrB1:204342744    3947 ---------- ---------- ---------- ---------- ---------- ---------- ---------- ---------- ----------  3947
ChrB4:45570261     3947 ---------- ---------- ---------- ---------- ---------- ---------- ---------- ---------- ----------  3947
enFeLV-clone4      3018 .......... .......... .......... .......... .......... .......... .......... .......... ..........  3107

enFeLV-AGTT        7268 AAAAGACACA AAAAGGACAT AAAGGGACTC ACTATCTAGC AGCCCCTAGC GGTACCTACT GGGCATGCAA CACCGGGCTA ACCCCATGCA  7357
enFeLV-clone1      7257 .C........ .......... .......... .......... .......... .......... .......... .......... ..........  7346
ChrB1:204342744    3947 ---------- ---------- ---------- ---------- ---------- ---------- ---------- ---------- ----------  3947
ChrB4:45570261     3947 ---------- ---------- ---------- ---------- ---------- ---------- ---------- ---------- ----------  3947
enFeLV-clone4      3108 .C........ .......... .......... .......... .......... .......... .......... .......... ..........  3197

enFeLV-AGTT        7358 TCTCCATGGC AGTGCTCAAC TGGACCTCTG ATTTTTGTGT CCTGATCGAG TTATGGCCCA GAGTAACTTA CCATGAGCCT GAGTATATCT  7447
enFeLV-clone1      7347 .......... .......... .......... .......... .......... .......... .......... .......... ..........  7436
ChrB1:204342744    3947 ---------- ---------- ---------- ---------- ---------- ---------- ---------- ---------- ----------  3947
ChrB4:45570261     3947 ---------- ---------- ---------- ---------- ---------- ---------- ---------- ---------- ----------  3947
enFeLV-clone4      3198 .......... .......... .......... .......... .......... .......... .......... .......... ..........  3287

enFeLV-AGTT        7448 ATTCCCACTT CGAAAATAAG CCCCGGTTCA AGAGAGATCC AATCTCCTTG ACAGTTGCCC TCATGCTGGG AGGAATCACT GTAGGGGGCA  7537
enFeLV-clone1      7437 .......... .......... .......... .......... .......... .......... .......... .......... ..........  7526
ChrB1:204342744    3947 ---------- ---------- ---------- ---------- ---------- ---------- ---------- ---------- ----------  3947
ChrB4:45570261     3947 ---------- ---------- ---------- ---------- ---------- ---------- ---------- ---------- ----------  3947
enFeLV-clone4      3288 .......... .......... .......... .......... .......... .......... .......... .......... ..........  3377

enFeLV-AGTT        7538 TGGCCGCCGG AATAGGAACA GGGACTGCGG CCCTTCTCGA GACAGCCCAA TTTAGACAAC TGCAAATGGC CATGCACACG GACATTCAGG  7627
enFeLV-clone1      7527 .......... .......... .......... .......... .......... .......... .......... .......... ..........  7616
ChrB1:204342744    3947 ---------- ---------- ---------- ---------- ---------- ---------- ---------- ---------- ----------  3947
ChrB4:45570261     3947 ---------- ---------- ---------- ---------- ---------- ---------- ---------- ---------- ----------  3947
enFeLV-clone4      3378 .......... .......... .......... .......... .......... .......... .......... .......... ..........  3467

enFeLV-AGTT        7628 CCTTAGAGGA GTCAATTAGT GCCTTAGAAA AATCCTTGAC TTCCCTTTCT GAAGTAGTCT TACAAAACAG GCGAGGCCTA GATATTCTGT  7717
enFeLV-clone1      7617 .......... .......... .......... .......... .......... .......... .......... .......... ..........  7706
ChrB1:204342744    3947 ---------- ---------- ---------- ---------- ---------- ---------- ---------- ---------- ----------  3947
ChrB4:45570261     3947 ---------- ---------- ---------- ---------- ---------- ---------- ---------- ---------- ----------  3947
enFeLV-clone4      3468 .......... .......... .......... .......... .......... .......... .......... .......... ..........  3557

enFeLV-AGTT        7718 TCCTACAGGA GGGAGGGCTA TGTGCAGCGT TAAAAGAAGA ATGTTGTTTT TATGCAGATC ACACCGGATT AGTCCGAGAT AATATGGCTA  7807
enFeLV-clone1      7707 .......... .......... .......... .......... .......... .......... .......... .......... ..........  7796
ChrB1:204342744    3947 ---------- ---------- ---------- ---------- ---------- ---------- ---------- ---------- ----------  3947
ChrB4:45570261     3947 ---------- ---------- ---------- ---------- ---------- ---------- ---------- ---------- ----------  3947
enFeLV-clone4      3558 .......... .......... .......... .......... .......... .......... .......... .......... ..........  3647

enFeLV-AGTT        7808 AATTAAGAGA AAGACTAAAA CAGCGACAAC AACTATTTGA CTCCCAGCAG GGATGGTTTG AAGGGTGGTT CAACAAGTCC CCCTGGTTCA  7897
enFeLV-clone1      7797 .......... .......... .......... .......... .......... .......... .......... .......... ..........  7886
ChrB1:204342744    3947 ---------- ---------- ---------- ---------- ---------- ---------- ---------- ---------- ----------  3947
ChrB4:45570261     3947 ---------- ---------- ---------- ---------- ---------- ---------- ---------- ---------- ----------  3947
enFeLV-clone4      3648 .......... .......... .......... .......... .......... .......... .......... .......... ..........  3737
```

Figure S15-12

```
enFeLV-AGTT        7898 CTACCTTAAT ATCCTCCATC ATGGGCCCCT TAATGATCCT CCTCCTAATT TTACTCTTCG GACCTTGCAT CCTTAACCGG TTGGTGCAGT  7987
enFeLV-clone1      7887 .......... .......... .......... .......... .......... ...T...... .......... .......... ..........  7976
ChrB1:204342744    3947 ---------- ---------- ---------- ---------- ---------- ---------- ---------- ---------- ----------  3947
ChrB4:45570261     3947 ---------- ---------- ---------- ---------- ---------- ---------- ---------- ---------- ----------  3947
enFeLV-clone4      3738 .......... .......... .......... .......... .......... .......... .......... .......... ..........  3827

enFeLV-AGTT        7988 TTGTCAAAGA TAGAATATCC GTCGTGCAGA CTTTAGTGCT AACCCAGCAA CACCAACGCC TGGGGCAATG CGACTCAGAC CAACCGTATC  8077
enFeLV-clone1      7977 .......... .......... .......... .......... .......... .......... .......... .......... ..........  8066
ChrB1:204342744    3947 ---------- ---------- ---------- ---------- ---------- ---------- ---------- ---------- ----------  3947
ChrB4:45570261     3947 ---------- ---------- ---------- ---------- ---------- ---------- ---------- ---------- ----------  3947
enFeLV-clone4      3828 .......... .......... .......... .......... .......... .......... .......... .......... ..........  3917

                           env                                                 3'LTR
enFeLV-AGTT        8078 ACCCCAGCTA AATGTATGAT TCCATTTAGG CTCCTAAGAA AAGGGGGAAA TGAAAGACCC CTTCCCCTTG TTTTGACCCC CTGTCATAAT  8167
enFeLV-clone1      8067 .......... .......... .......... .......... .......... .......... .......... .......... ..........  8156
ChrB1:204342744    3947 --........ .......... ...G...... .......... .......... ......T... .......... C......... ..........  4035
ChrB4:45570261     3947 --........ .......... .......... .......... .......... ......T... .......... C......... ..........  4035
enFeLV-clone4      3918 .......... .......... .......... .......... .......... .......... .......... .......... ..........  4007

enFeLV-AGTT        8168 ATGCTTAGCA ATAGTAACGC CATTTGCAAG ACAGCACCAA GAAGTTCAGG GGTCTTATCC TAAGTCCACC GTTTAGCTGC CAAACAGGAT  8257
enFeLV-clone1      8157 .......... .......... .......... .......... .......... .......... .......... .......... ..........  8246
ChrB1:204342744    4036 .......... .......... .......... G........G .......... .........T ......A... .......... ..........  4125
ChrB4:45570261     4036 .......... .......... .......... G........G .......... .........T .....GA... .......... .......TG.  4125
enFeLV-clone4      4008 .......... .......... .......... .......... .......... .......... .......... .......... ..........  4097

enFeLV-AGTT        8258 ATCTGTGGTC AGCCACCCGG CCCTAAGATA GCCACCTGGC CCTAAGATGG GAATGGAAAG TACTGACTCC ACCCGATAGA CCCTAGAGAT  8347
enFeLV-clone1      8247 .......... .......... .......... .......... .......... .......... .......... .......... ..........  8336
ChrB1:204342744    4126 .......... .......T.. .......... .GA..GGA.A GTACT..CTC C.CCC..T.- ...CCTAGAG .TGA.CC.TG T.AGCC.TCC  4214
ChrB4:45570261     4126 .......... .......T.. .......... .GA..GGAAA GTACT..CTC C.CCC..T.- ...CCTAGAG .TGA.CCT.G T.AGCC.CCC  4214
enFeLV-clone4      4098 .......... .......... .......... .......... .......... .......... .......... .......... ..........  4187

enFeLV-AGTT        8348 GAGCCTAGTC AGC-CACCCA TGTTTTTCCC CCTCATTCTG GGAAATCGCC CTCAGAAAAG AAAAGAAAAA GAAAAAAAAA AAAAAAAAAA  8436
enFeLV-clone1      8337 .......... ...-...... .......... .......... .......... .......... .....----- ---------. ..........  8411
ChrB1:204342744    4215 AT.TT.GT.. CC.T..TT.T G.AAAA..A. .....GG--- ---------- ---------- ---------- ---------- --------..  4253
ChrB4:45570261     4215 AT.TT.GT.. CC.T..TT.T G.AAAA..A. .....GG--- ---------- ---------- ---------- ---------- --------..  4253
enFeLV-clone4      4188 .......... ...-...... .......... .......... .......... .......... .....----- -------.G. ..........  4264

enFeLV-AGTT        8437 AAAAAACCAG CCTCATTTAA CTGGACCAAT AAG-ACCCCG TAACTATGCT TCTCGCTTCT GTAACCGCGC TTCTGCCACT CCAACCCTAT  8525
enFeLV-clone1      8412 .......... .......... .......... ...-...... .......... .......... .......... .......... ..........  8500
ChrB1:204342744    4254 .......... .......... .......... ...-...... ....C..... .......... .......... .......... ..........  4342
ChrB4:45570261     4254 .......... .......... A......... ...A...... ....C..... .......... .......... .......... ..........  4343
enFeLV-clone4      4265 .......... .......... .......... ...-...... .......... .......... .......... .......... ..........  4353

enFeLV-AGTT        8526 AAAAAGTCTC CCCAGCCCAA CAAGAGGCGC GCAAGTCTTT GCTGAGACTT GACCGCCCCG GGTACCCGTG TA-CGAATAA ACCTCTTGCT  8614
enFeLV-clone1      8501 .......... ....A..... .......... .......... .......... .......... .......... ..-....... ..........  8589
ChrB1:204342744    4342 -......... .......... ..G....... .......... .......... .......... .......... ..C....... ..........  4431
ChrB4:45570261     4343 -......... .......... ..G....... .......... .......... .......... .......... ..C....... ..........  4432
enFeLV-clone4      4354 .......... .......... .......... .......... .......... .......... .......... ..-....... ..........  4442
```

Figure S15-13

```
enFeLV-AGTT         8615 GTTTGCATCT GACTCGTGGT CTCGGTGTTC CGTGGGCACG GGGTCTCATC GCCGAGGAAG ACCTAGTTAG GGGGTCTTTC A 8695
enFeLV-clone1       8590 .......... .......... .......... .......... .......... .......... ........C. .......... . 8670
ChrB1:204342744     4432 .......... .......... .......... .......... .......... .......... ........CT .......... . 4512
ChrB4:45570261      4433 .......... .......... .......... .......... .......... .......... ........CT .......... . 4513
enFeLV-clone4       4443 .......... .......... .......... .......... .......... .......... ........C. .......... . 4523
```

Table S1. Sequences of primers used in this study

Primer	Sequence (5'– 3')
enFeLVC4	ACCTGGATCCGCCGCCACCATGGAAGGTCCAACGCACCC
RBD-F1	ACCTGGATCCGCCGCCACCATGGAAAGTCCAACGCACCC
FeLIX-1F	CTGGATCCATGGAAGGTCCAACGCACCCAA
FeLIX-P	TCCCCTCATTCTGGAAAATCACCCTCAGGA
FeLIX-F	CACCCGATATACCCTAGAGATGAG
Fe-36S	AACCGCTTGGTACARTTCATAAGAG
Fe-217S	ATCCGGATCCATGGAAGGTCCAACGCACCCAAAA
Fe-227S	GTCAACCCCATCGTGTTTTT
Fe-265S	AATTGCTGGCGAGGAAGTTATC
Fe-360S	TCACTATGGAGGGGTGATGTTG
Fe-362S	TTCTTGCTGCCTATTGTCTGGA
Fe-361S	CTCAGGTGCCCCTAAAAAGAGA
Fe-431S	GCTTGTGAGATGTGGGTACAGC
Fe-456S	GGTAATGGACATCCAAGGTTCC
Fe-457S	AAGGTAAGGTTTTCCCCCACAT
Fe-560S	GCCGAATTCGCCGCCCACCATGGAAGGTCCAACGCACCCAAAACCCTCT
Fe-710S	GACATCAAACCAAGAACCTCGA
Fe-711S	GGATCCACGCCTCTCATGTA
Fe-720S	GCCCCTGGTTTTATACCGGGTACGTA
Fe-721S	AGCTCAGACGATCCATCGAGATGGAAGGTCCAACGCACCC
Fe-761S	CAGCCTATGAGGAGGTGGCGACAGAGAAACACACCCTTT
Fe-766S	ACCTGGATCCGCCGCCACCATGGTATTGCTGCCTGGGTC
Fe-792S	CAGCCTATGAGGAGGTGGCAACAGAGAAACACACCCTTT
Fe-782S	TCCCAGGGTATGGATGTGATCAGCCTATGAGGAGGTG
Fe-844S	CAATCAGGAAGGGCTTGATC
Fe-845S	GACAGTAGAAACACTAATGG
Fe-861S	CTTGGGATGGACCTAAGTCATGG
Fe-862S	ACCAATCAGGACCCCTCAGA
Fe-60R	GAGTCTTATTTGCATACAGGCTGGT
Fe-204R	CTGCTGTCTTGGGAACTTTGTC
Fe-237R	CGGCCTGCTTATCTGACTCTTC
Fe-397R	CTCAGGCACTTCTAGCTGCTCA
Fe-400R	CCTGACGCAAAAAGAGTCTCAA
Fe-399R	TCAGCAACTCTCTGTTAATCTTTGG
Fe-466R	AAGCACCGGGCTAATTTAGAGA
Fe-448R	AACACATCAAAGCACACCTCGT
Fe-475R	CAGAGGAAGTGGGGAAGAAAGA
Fe-550R	GGCGCTAGCCTAAATGGAATCATACATTTAGCTGG
Fe-554R	CCAGTGCCAAGCTTGCATGCCTGCA
Fe-686R	TGCAGAATTCTCATGTTGTGGGTCGGATAGGCA
Fe-716R	TGCAGAATTCTCACAGATCCTCCTCGGAGATCAGCTTCTGCTCGGCCTGAGGCGGCGTAATGG
Fe-730R	ATGGGCAAGACAGCTTGGTT
Fe-747R	GGGTGCGTTGGACCTTCCATCTCGATGGATCGTCTGAGCT
Fe-748R	ATCATACATTTAATTGGAAATTAGCTGGGGTGATACGGTT
Fe-750R	CGGCCTGCTTATCTGACTCT
Fe-788R	AAAGGGTGTGTTTCTCTGTCGCCACCTCCTCATAGGCTG
Fe-790R	TGCAGAATTCTCAAAGGTTACCTTCGTTCTCTA
Fe-807R	CACCTCCTCATAGGCTGATCACATCCATACCCTGGGA
Fe-812R	AAAGGGTGTGTTTCTCTGTTGCCACCTCCTCATAGGCTG
Fe-874R	CCACACAGCAGAACCAAACAT
Fe-875R	CTCAGGAAGGATGCAATGAG
Fe-891R	TAGCTGGGGTGAGGTATTAC
Fe-892R	GCTTAACCAACGGAGTCCCA
FeLIX-1R	GAAGCGAGAAGCATGGTTACG
SDM-FeLIX1	TCAGGCCATGGGACCAAATCCAGTCCTGCCTGATC
SDM-FeLIX2	GATCAGGCAGGACTGGATTTGGTCCCATGGCCTGA

Table S2. Characteristic of enFeLV in domestic cat used in this study

Name	Chromosome: provirus position	Chromosome: *env* position	ORF	Length of amino acids (Env)	Age (Mya)	Accession number	Source
enFeLV-clone1	ChrB4: 83161862..83153192	ChrB4:83168073..83170071	Intact *env*	666	0.2166	LC196053.1	In this study
enFeLV-clone2	ChrB3:77504220..77495524	ChrB3:77510006..77512004	Intact *env*	666	0.1062	LC196054.1	In this study
enFeLV-clone3	ChrA2:158856703..158865693	ChrA2:158862891..158864892	Intact *env*	666	0	LC196055.1	In this study
enFeLV-clone4	ChrB4:63249450..63253973	ChrB4:63247526..63246420	Truncated *env*	369	0.2166	LC198317.1	In this study
enFeLV-clone5	ChrB4:45466253..45466874	ChrB4:45469880..45470699	Truncated *env*	273	0.696	LC198318.1	In this study
enFeLV-clone6	ChrA3:22964466..22967845	ChrA3:22966277..22967249	Truncated *env*	90	0.3294	LC198319.1	In this study
enFeLV-AGTT	ChrA1: 205669512..205679407	ChrA1:205676200..205678198	Intact *env*	666	0	AY364318.1	Isolate
enFeLV-GGAG	ChrA2: 69618599..69628466	ChrA2:69625273..69626377	Truncated *env*	368	0.11	AY364319.1	Isolate
enFeLV-CFE6	ND	ND	Intact *env*	668	0.2334	M25425.1	Isolate
enFeLV-CFE16	ND	ND	Truncated *env*	273	0.4668	M25582.1	Isolate
Fca strain C3	ND	ND	Truncated *env*	450	ND	OP595720.1	Isolate
Fca strain C12	ND	ND	Intact *env*	666	0	OP595706.1	Isolate
Fca strain C16	ND	ND	Truncated *env*	514	0	OP595709.1	Isolate
Fca strain C30	ND	ND	Intact *env*	666	0.5574	OP595707.1	Isolate
Fca strain C31	ND	ND	Truncated *env*	469	ND	OP595714.1	Isolate
Fca strain C33	ND	ND	Truncated *env*	437	ND	OP595716.1	Isolate
Fca strain C34	ND	ND	Intact *env*	666	0	OP595708.1	Isolate
ChrA1:237978323	ChrA1:237978323..237983035	ChrA1:237981730..237982045	Truncated *env*	48	0	CM028198	Cat genome (NCBI)
ChrA2:126590453	ChrA2:126590453..126599114	ChrA2:126596525..126598523	Intact *env*	666	0	AP023153	Cat genome (NCBI)
ChrB1:153791216	ChrB1:153791216..153795411	ChrB1:153791714..153792041	Truncated *env*	48	0	CM031415	Cat genome (NCBI)
ChrB1:204333001	ChrB1:204333001..204356576	ChrB1:204345879..204346698	Truncated *env*	273	0.4644	NC_058371	Cat genome (NCBI)
ChrB2:29977771	ChrB2:29977771..29986443	ChrB2:29983847..29984951	Truncated *env*	368	0	CM031416	Cat genome (NCBI)
ChrB2:3162779	ChrB2:3162779..3171382	ChrB2:3168829..3176877	Intact *env*	666	0	CM031416	Cat genome (NCBI)
ChrB3:137955648	ChrB3:137955648..137964319	ChrB3:137961723..137963721	Intact *env*	666	0	CM028203	Cat genome (NCBI)
ChrB4:1815451	ChrB4:1815451..1824152	ChrB4:1821542..1823540	Intact *env*	666	0	CM031418	Cat genome (NCBI)
ChrB4:83104800	ChrB4:83104800..83113469	ChrB4:83110877..83112875	Intact *env*	666	0.2166	CM031418	Cat genome (NCBI)
ChrB4:45570261	ChrB4:45570261..45574773	ChrB4:45577391..45578210	Truncated *env*	273	0.4632	CM031418	Cat genome (NCBI)
ChrB4:29885536	ChrB4:29885536..29894216	ChrB4:29894159..29894411	Truncated *env*	84	0.3258	CM028204	Cat genome (NCBI)
ChrB4:82927705	ChrB4:82927705..82931318	ChrB4:82929648..82930302	Truncated *env*	218	ND	CM028204	Cat genome (NCBI)
ChrB4:85235849	ChrB4:85235849..85244527	ChrB4:85236446..85238444	Intact *env*	666	0.654	CM001384	Cat genome (NCBI)
ChrB4:47523766	ChrB4:47523766..47528223	ChrB4:47527257..47527386	Truncated *env*	43	0.207	CM001384	Cat genome (NCBI)
ChrB4:47520004	ChrB4:47520004..47524419	ChrB4:47522893..47523331	Truncated *env*	146	0.69	CM001384	Cat genome (NCBI)
ChrD1:109672515	ChrD1:109672515..109679862	ChrD1:109673547..109673604	Truncated *env*	19	ND	CM001387	Cat genome (NCBI)
ChrD1:700129	ChrD1:700129..703822	ChrD1:700870..701017	Truncated *env*	49	ND	CM028207	Cat genome (NCBI)
ChrD3:26685272	ChrD3:26685272..26688966	ChrD3:26687913..26688060	Truncated *env*	49	ND	CM028209	Cat genome (NCBI)
ChrD4:68171	ChrD4:68171..76919	ChrD4:70243..70263	Truncated *env*	20	2.064	CM001390	Cat genome (NCBI)
ChrD4:92824	ChrD4:92824..101492	ChrD4:93422..95420	Intact *env*	666	0	CM028210	Cat genome (NCBI)
ChrF1:11537581	ChrF1:11537581..11543055	ChrF1:11541748..11541865	Truncated *env*	39	6	CM001394	Cat genome (NCBI)
ChrF1:9624604	ChrF1:9624604..9629638	ChrF1:9628585..9628732	Truncated *env*	49	ND	CM028214	Cat genome (NCBI)
ChrX:8830722	ChrX:8830722..8839611	ChrX:8832832..8833393	Truncated *env*	187	3.402	CM001396	Cat genome (NCBI)

ND: not determined due to not complete of sequence or not available

Table S3. The estimated of integration timing based on the substitution rate of 5'LTR and 3'LTR sequences

Provirus name	LTR length (bp)		Pairwise Genetic Distance	Calculated integration age (thousand years ago)
	5`LTR	3`LTR		
enFeLV-clone1	556	554	0.00361	216.6
enFeLV-clone2	573	564	0.00177	106.2
enFeLV-clone3	563	565	0	Recently
enFeLV-clone4	554	556	0.00361	0.2166
enFeLV-clone5	516	518	0.0116	696
enFeLV-clone6	516	555	0.00549	329.4
enFeLV-AGTT	568	568	0	Recently
enFeLV-GGAG	554	553	0.00181	108.6
enFeLV-CFE6	514	516	0.00389	233.4
enFeLV-CFE16	515	517	0.00778	466.8
Fca strain C3	Absent	543	ND	ND
Fca strain C12	542	543	0	Recently
Fca strain C16	542	542	0	Recently
Fca strain C30	538	541	0.00929	557.4
Fca strain C31	Absent	541	ND	ND
Fca strain C33	Absent	542	ND	ND
Fca strain C34	538	538	0	Recently
ChrA1:237978323	542	543	0	Recently
ChrA2:126590453	552	551	0	Recently
ChrB1:153791216	542	542	0	Recently
ChrB1:204333001	520	517	0.00774	464.4
ChrB2:29977771	556	557	0	Recently
ChrB2:3162779	517	516	0	Recently
ChrB3:137955648	556	558	0	Recently
ChrB4:1815451	571	572	0	Recently
ChrB4:83104800	556	554	0.00361	216.6
ChrB4:45570261	519	521	0.00772	463.2
ChrB4:29885536	555	568	0.00543	325.8
ChrB4:82927705	Absent	555	ND	ND
ChrB4:85235849	584	554	0.0109	654
ChrB4:47523766	654	292	0.00345	207
ChrB4:47520004	262	515	0.0115	690
ChrD1:109672515	268	289	0.527	ND
ChrD1:700129	268	289	0.525	ND
ChrD3:26685272	268	289	0.527	ND
ChrD4:68171	555	562	0.0344	2064
ChrD4:92824	554	555	0	Recently
ChrF1:11537581	538	520	0.1	6000
ChrF1:9624604	268	289	0.527	ND
ChrX:8830722	615	598	0.0567	3402

ND: not determined due to not complete of LTR sequence
